# Perturbations in imprinted methylation from assisted reproductive technologies but not advanced maternal age in mouse preimplantation embryos

**DOI:** 10.1186/s13148-019-0751-9

**Published:** 2019-11-26

**Authors:** Audrey J. Kindsfather, Megan A. Czekalski, Catherine A. Pressimone, Margaret P. Erisman, Mellissa R. W. Mann

**Affiliations:** 1 Department of Obstetrics, Gynecology and Reproductive Sciences, University of Pittsburgh School of Medicine, 204 Craft Ave, Pittsburgh, PA 15213 USA; 2 Magee-Womens Research Institute, 204 Craft Ave, Pittsburgh, PA 15213 USA

**Keywords:** Genomic imprinting, DNA methylation, Fertility, Assisted reproductive technologies, Maternal age, Mouse

## Abstract

**Background:**

Over the last several decades, the average age of first-time mothers has risen steadily. With increasing maternal age comes a decrease in fertility, which in turn has led to an increase in the use of assisted reproductive technologies by these women. Assisted reproductive technologies (ARTs), including superovulation and embryo culture, have been shown separately to alter imprinted DNA methylation maintenance in blastocysts. However, there has been little investigation on the effects of advanced maternal age, with or without ARTs, on genomic imprinting. We hypothesized that ARTs and advanced maternal age, separately and together, alter imprinted methylation in mouse preimplantation embryos. For this study, we examined imprinted methylation at three genes, *Snrpn*, *Kcnq1ot1*, and *H19*, which in humans are linked to ART-associated methylation errors that lead to imprinting disorders.

**Results:**

Our data showed that imprinted methylation acquisition in oocytes was unaffected by increasing maternal age. Furthermore, imprinted methylation was normally acquired when advanced maternal age was combined with superovulation. Analysis of blastocyst-stage embryos revealed that imprinted methylation maintenance was also not affected by increasing maternal age. In a comparison of ARTs, we observed that the frequency of blastocysts with imprinted methylation loss was similar between the superovulation only and the embryo culture only groups, while the combination of superovulation and embryo culture resulted in a higher frequency of mouse blastocysts with maternal imprinted methylation perturbations than superovulation alone. Finally, the combination of increasing maternal age with ARTs had no additional effect on the frequency of imprinted methylation errors.

**Conclusion:**

Collectively, increasing maternal age with or without superovulation had no effect of imprinted methylation acquisition at *Snrpn*, *Kcnq1ot1*, and *H19* in oocytes. Furthermore, during preimplantation development, while ARTs generated perturbations in imprinted methylation maintenance in blastocysts, advanced maternal age did not increase the burden of imprinted methylation errors at *Snrpn*, *Kcnq1ot1*, and *H19* when combined with ARTs. These results provide cautious optimism that advanced maternal age is not a contributing factor to imprinted methylation errors in embryos produced in the clinic. Furthermore, our data on the effects of ARTs strengthen the need to advance clinical methods to reduce imprinted methylation errors in in vitro-produced embryos.

## Introduction

Recent societal trends have resulted in women delaying pregnancy until later in their childbearing years. As such, over the last several decades, maternal age at the first and subsequent pregnancies has risen steadily [1]. Notably, birth rates have significantly increased among women 35–55 years of age, which is considered advanced maternal age, labeling the current decade as an “epidemic of age-related infertility” [1–3]. This increase in maternal age has contributed in part to the tripling of infertility rates since 1984 (~ 5% to 15%) [4, 5]. After the age of 35, a woman’s fertility declines, and in the likelihood that a pregnancy is achieved, the risk of adverse outcomes increases [6, 7]. Thus, it is crucial to understand the molecular consequences of advanced maternal age in gametes and embryos.

One effect of aging is nuclear changes in DNA methylation. A landmark paper in 2005 discovered that as monozygotic twins age, their DNA methylomes become increasingly different [8]. Subsequent studies found that age-related changes in DNA methylation accumulate over a lifetime [9–11]. In females, reproductive or oocyte aging is defined as a progressive decline in oocyte numbers and quality [12, 13]_._ To date, only a couple of studies have investigated DNA methylation or the catalytic enzymes involved, DNA methyltransferases (DNMTs), in aged oocytes or the resulting embryos. Advanced maternal age leads to alterations in gene expression in oocytes, including *Dnmt1* and *Dnmt3b*, as well as a decrease in DNMT1, DNMT3A, and DNMT3B protein levels [14, 15]. Furthermore, these oocytes produced embryos with a decrease in global DNA methylation during preimplantation embryo development [14].

Genomic imprinting is another epigenetic mechanism that employs DNA methylation. Here, one copy of a gene is silenced based on the parental origin of inheritance, while the other parental allele is expressed [16]. These sex-specific DNA methylation marks are acquired during gametogenesis and maintained during preimplantation development [17, 18]. To date, the effects of advanced maternal age on the acquisition and maintenance of imprinted DNA methylation in oocytes and resulting preimplantation embryos, respectively, have not been investigated.

In response to the age-related fertility decline, women of advanced maternal age frequently turn to ARTs as medical treatments for subfertility, which often represent the best recourse for achieving a pregnancy. While generally considered safe, ARTs, including hormone-induced ovarian hyperstimulation (superovulation) and in vitro embryo culture, have been linked to imprinting disorders. Significantly, there are more cases of Beckwith-Wiedemann syndrome (BWS), Silver-Russell syndrome (SRS), Angelman syndrome (AS), and Prader-Willi syndrome (PWS) due to imprinted methylation errors in children conceived with ARTs compared to the general population, suggesting that imprinting defects are exacerbated by ARTs [19–35]. This is supported by our published findings, which have demonstrated that ARTs lead to imprinted methylation errors in early mouse and likely human embryos from young mothers [36–42], although imprinted methylation acquisition in mouse oocytes is not altered by superovulation [43]. However, there has been little investigation on the effects of advanced maternal age in combination with ARTs on genomic imprinting.

In this study, we hypothesized that advanced maternal age leads to perturbations in imprinted methylation acquisition in oocytes and/or imprinted methylation maintenance in blastocysts. To facilitate this analysis, as well as relate our findings to maternal age in humans, we produced a model of maternal aging, extending from puberty to reproductive senescence, that more precisely relates human and mouse ages [44, 45]. Since imprinted gene regulation is well conserved between mouse and human [18, 46], and imprinting disorders in ART-conceived children are associated with methylation perturbations at *SNRPN*, *KCNQ1OT1*, and *H19* [19–35], we investigated imprinted methylation at *Snrpn*, *Kcnq1ot1*, and *H19* in our mouse model. We also hypothesize that a combination of advanced maternal age with ARTs results in a higher frequency of blastocysts with a loss of imprinted methylation than any ART alone. Prior to addressing this hypothesis, we first characterized the effects of various ARTs on imprinted methylation maintenance in mouse blastocysts. More specifically, we examined superovulation and embryo culture, singly and together, and with respect to the latter, we also investigated low and ambient oxygen conditions, since low oxygen has been recommended based on improved embryo development [47–50]. We found that imprinted methylation acquisition and maintenance were unaffected by increasing maternal age, and that the frequency of imprinted methylation errors in blastocysts was unchanged when increasing maternal age was combined with ARTs. In conclusion, while a combination of ARTs altered imprinted methylation at *Snrpn*, *Kcnq1ot1*, and *H19* in mouse blastocysts, advanced maternal age did not increase the burden of imprinted methylation errors when combined with ARTs.

## Results

### Imprinted methylation acquisition in oocytes was unaltered by advanced maternal age

**Fig. 1:**
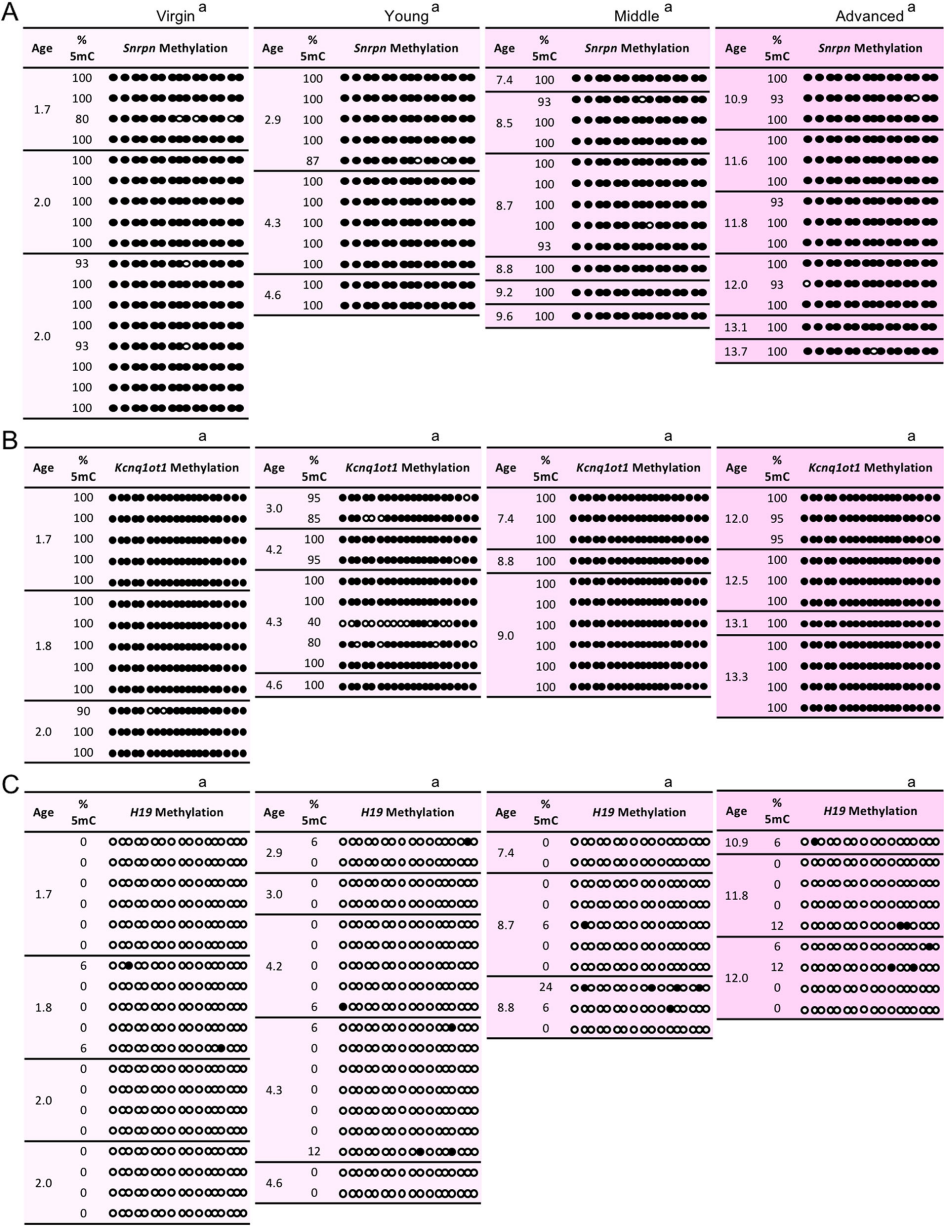
Imprinted methylation acquisition at the (**a**) *Snrpn*, (**b**) *Kcnq1ot1*, and (**c**) *H19* ICRs in germinal vesicle oocytes was unaltered by increasing maternal age. Each box shows oocytes from spontaneously ovulating females with maternal age in months (mths) (*n* = 3–4 females per age group). Each line of circles represents the CpGs in the *Snrpn*, *Kcnq1ot1*, or *H19* ICRs in an individual oocyte (*n* = 13–19 oocytes per age group). Oocytes were excluded if sequenced DNA clones (*n* = 5–8/denuded oocyte) exhibited more than one methylation pattern, suggestive of cumulus cell contamination. Black circles, methylated CpGs; white circles, unmethylated CpGs; %5mC, percentage of 5-methyl CpGs over the total number of CpGs for a specific ICR. Same letter (a), no statistically significant difference between groups

During gamete development, extensive genome-scale epigenetic transitions occur, including erasure of DNA methylation in primordial germ cells and subsequent acquisition of sex-specific imprinted methylation marks [17, 18]. Given that studies showed that advanced maternal age leads to changes in gene expression and protein levels of DNA methyltransferases [14, 15], the first hypothesis we tested was whether advanced maternal age, without ARTs, altered the ability of oocytes to normally acquire imprinted methylation marks. To address this, germinal vesicle (GV) oocytes from spontaneously ovulating female mice in four maternal age groups were examined for their imprinted methylation status at the *Snrpn*, *Kcnq1ot1*, and *H19* imprinting control regions (ICRs), which are associated with AS, PWS, BWS, and SRS. Any perturbation in the acquisition of imprinted methylation would be apparent by this late preovulatory stage of oogenesis [51]. Female mice were categorized based on a model we produced for maternal aging that more precisely relates human and mouse ages [44, 45]. Young maternal age in mice of > 2–6 months corresponded to humans of 15–25 years. Middle maternal age mice of > 6–10 months paralleled > 25–35 years in humans, while advanced maternal age of > 10–14 months in mice corresponded to > 35–45 years in humans (Additional file 1: Figure S1). Female mice in these three age groups were retired breeders with proven fertility. Virgin females between 1.5 to 2 months old served as controls. During oocyte growth, *Snrpn* and *Kcnq1ot1* acquire DNA methylation and by the germinal-vesicle and mature MII stages, this acquisition is complete [69, 71, 73–75]. By comparison, *H19* remains unmethylated in oocytes. Our analysis showed that, except for one oocyte from a young female with 40% *Kcnq1ot1* methylation, GV oocytes from all maternal age groups had the same high levels of *Snrpn* and *Kcnq1ot1* methylation and the same low levels of *H19* methylation as GV oocytes from virgin females (Fig. 1). These results indicate that imprinted methylation was acquired normally during oogenesis in females of advanced maternal age.

### Imprinted methylation acquisition was unchanged by advanced maternal age combined with superovulation

**Fig. 2:**
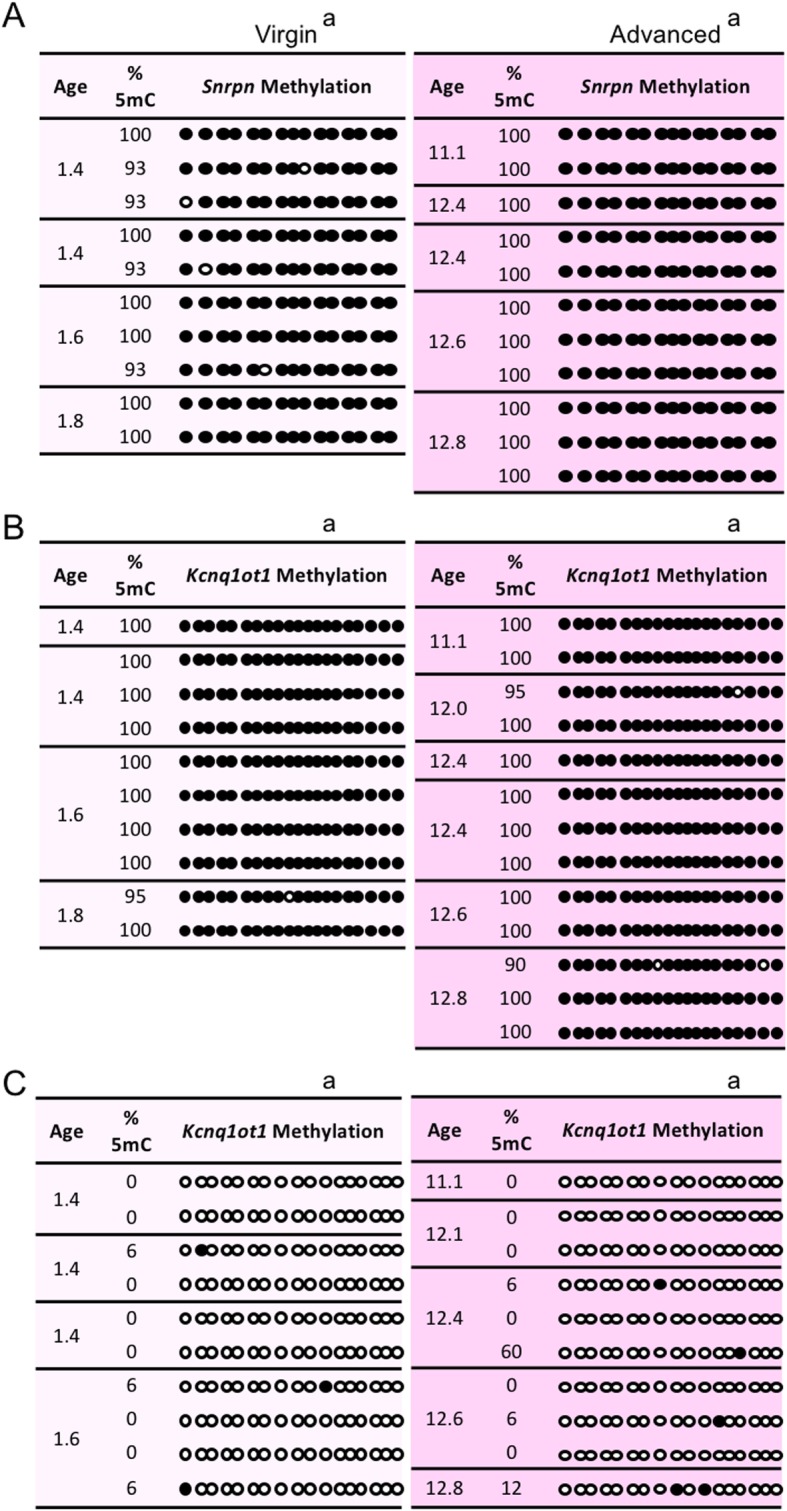
Imprinted methylation acquisition at the (**a**) *Snrpn*, (**b**) *Kcnq1ot1*, and (**c**) *H19* ICRs in MII oocytes was unchanged by increasing maternal age combined with superovulation. Each box shows oocytes from superovulated females with maternal age in months (mths) (*n* = 3–4 females per age group; *n* = 10–13 oocytes per age group). See Fig. 1 for details. Same letter (a), no statistically significant difference between groups

We previously showed that superovulation (SO) does not affect imprinted methylation acquisition as assessed in meiosis II (MII) oocytes from virgin females [43]. However, whether the combination of maternal age and SO impacts acquisition of imprinted methylation has not been investigated. To address this, MII oocytes from superovulated virgin (1.5–2 months old) and advanced maternal age (> 10 months old) females were assessed for imprinted methylation at the *Snrpn*, *Kcnq1ot1*, and *H19* ICRs. Compared to controls, MII oocytes from advanced-aged females displayed similar hypermethylation of the *Snrpn* and *Kcnq1ot1* ICRs and similar hypomethylation of the *H19* ICR (Fig. 2). These data indicate that imprinted methylation acquisition was not adversely affected by advanced maternal age plus SO, and are consistent with our previous published data on SO alone [43].

### Postzygotic imprinted methylation maintenance was unaltered by advanced maternal age

**Fig. 3:**
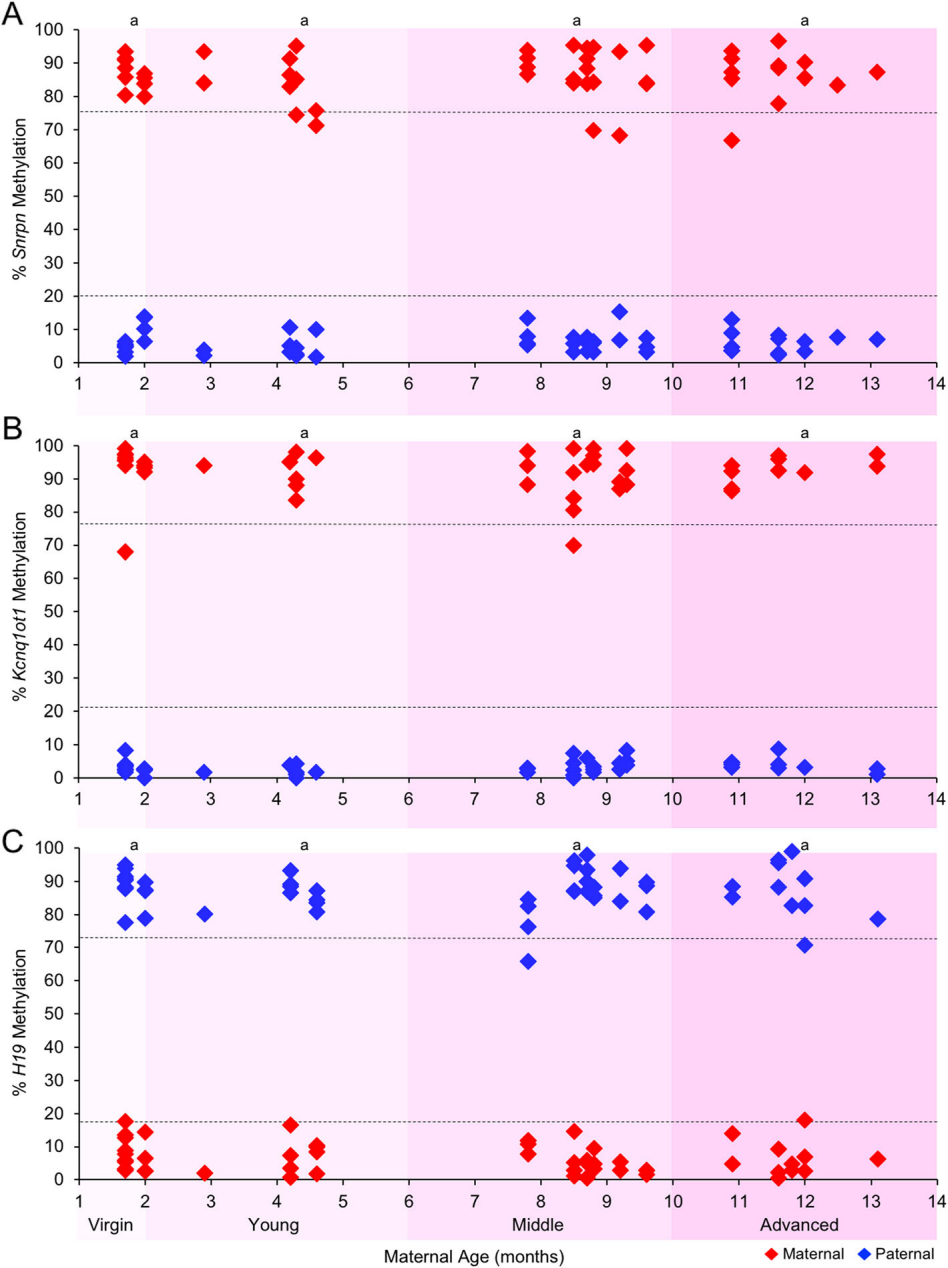
Advanced maternal age did not perturb imprinted methylation maintenance of the (**a**) *Snrpn*, (**b**) *Kcnq1ot1*, and (**c**) *H19* ICRs in blastocysts. Embryos were derived from spontaneously ovulating females. Diamonds represent the mean methylation levels of maternal (red) or paternal (blue) alleles for the *Snrpn*, *Kcnq1ot1*, and *H19* ICRs in individual blastocysts (*n* = 9–22 embryos, *n* = 3–7 females per age group). Same letter (a), no statistically significant difference between groups

Another important stage of global epigenetic alterations is preimplantation development, when genome-scale demethylation occurs [17, 18]. However, some genomic regions are protected from the demethylation machinery. These regions include ICRs, which maintain their allele-specific methylation patterns during this period. In previous studies, we found that while SO did not alter imprinted methylation acquisition in oocytes [43], it led to perturbations in imprinted methylation maintenance in preimplantation embryos [40]. Here, we tested the hypothesis that advanced maternal age leads to postzygotic changes in imprinted methylation during preimplantation development. Thus, blastocysts from spontaneously ovulating virgin, young, middle-, and advanced-aged females were assessed for *Snrpn*, *Kcnq1ot1*, and *H19* imprinted methylation. Similar to controls, blastocysts from females of increasing maternal age had no significant difference in imprinted methylation, i.e., the maternal *Snrpn*, maternal *Kcnq1ot1*, and paternal *H19* ICRs possessed normal hypermethylation (≥ 75% methylation), while the paternal *Snrpn*, paternal *Kcnq1ot1*, and maternal *H19* ICRs harbored normal hypomethylation (< 20% methylation) (Fig. 3; Additional file 1: Figures S2, S3, and S4). These results indicate that maintenance of imprinted methylation was not adversely affected by advanced maternal age.

As females age, they are naturally prone to weight gain [52]. Several studies in humans and mice have reported that maternal weight and nutrition prior to conception and during pregnancy alters DNA methylation in their offspring [53–55]. Thus, we questioned whether there was a relationship between maternal weight and imprinted methylation in blastocyst-stage embryos described above. While we observed a positive correlation between maternal age and weight, no association was found between maternal weight and imprinted methylation levels at the *Snrpn*, *Kcnq1ot1*, and *H19* ICRs in blastocysts (Additional file 1: Figure S5). This suggests that natural age-associated weight gain per se does not perturb maintenance of imprinted methylation in preimplantation embryos.

### Additive effects of ARTs on imprinted methylation errors in blastocysts

**Fig. 4:**
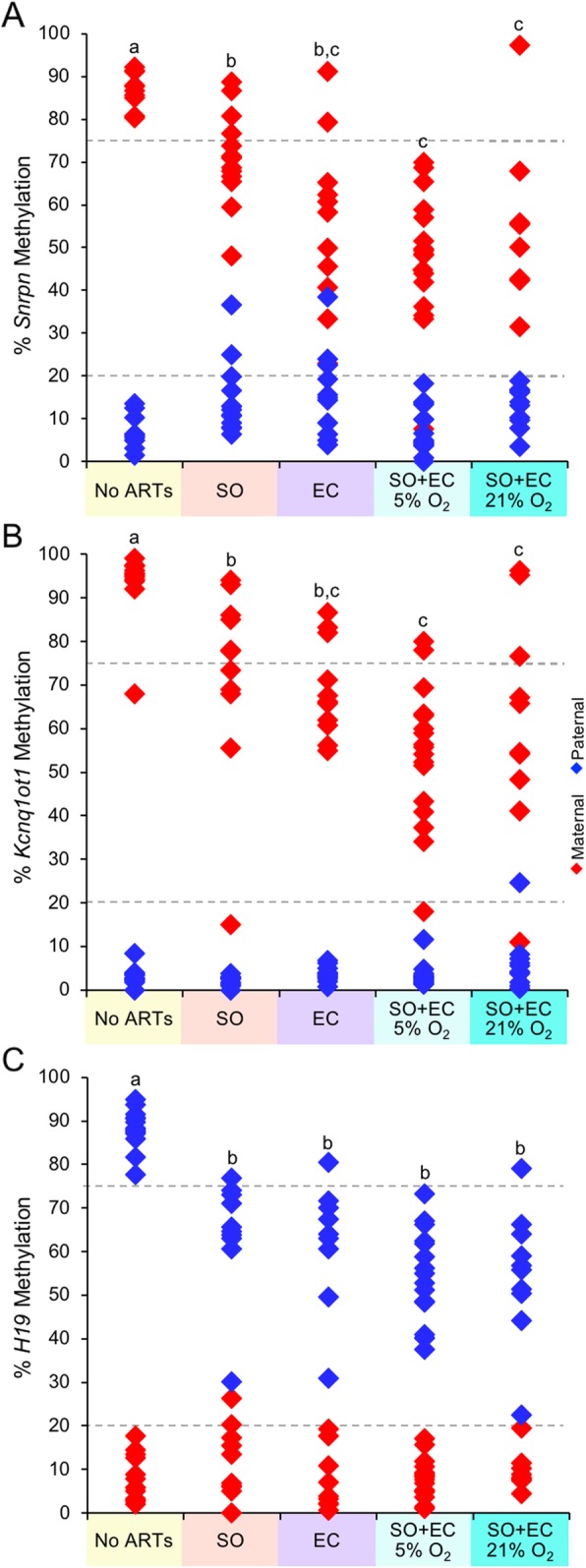
ARTs generated a loss of imprinted methylation maintenance at the (**a**) *Snrpn*, (**b**) *Kcnq1ot1*, and (**c**) *H19* ICRs in blastocysts from young mothers. Diamonds represent the mean methylation levels of maternal (red) or paternal (blue) alleles for *Snrpn*, *Kcnq1ot1*, and *H19* ICRs in individual embryos (*n* = 9–17 blastocysts; *n* = 3–4 females per treatment group). SO, superovulation; EC, embryo culture. Letters (a, b, c), a statistically significant difference between groups (Additional file 1: Table S1)

In a previous study, we reported that a combination of SO and embryo culture (EC) increased the frequency of embryos with a loss of *H19* imprinted expression when compared to embryos that were in vivo-derived or derived from SO alone [39]. A comprehensive study examining imprinted methylation comparing maternal age with and without ARTs has not previously been performed. Thus, prior to investigating the effects of maternal age with ARTs on imprinted methylation, we first characterized the effects of ARTs (SO only, EC only, and both SO+EC) on imprinted methylation maintenance in mouse blastocysts compared to no ARTs (spontaneous ovulation, in vivo derived) using virgin females. Compared to the no ARTs group, we observed a significant loss in imprinted methylation in blastocysts from the SO group, the EC group, as well as the SO+EC group at the maternal *Snrpn*, maternal *Kcnq1ot1*, and the paternal *H19* ICRs (Fig. 4; Additional file 1: Figures S6, S7, and S8; Additional file 1: Table S1).

A comparison between ART groups revealed no significant difference between the SO only and EC only groups for the frequency of blastocysts with imprinted methylation loss at the maternal *Snrpn*, maternal *Kcnq1ot1*, and paternal *H19* ICRs (Fig. 4; Additional file 1: Figures S6, S7, and S8; Additional file 1: Table S1). However, compared to SO alone, a significant increase in the frequency of imprinted methylation loss occurred at the maternal *Snrpn* and maternal *Kcnq1ot1* ICRs in blastocysts from the SO+EC groups, but not for the paternal *H19* ICR. With regard to EC, no significant difference was found between the EC only and SO+EC groups for the three imprinted genes.

Embryo culture under low oxygen conditions has been recommended since numerous studies utilizing low oxygen have reported improved embryo development [47–50]. However, few studies have examined the effects of low and high oxygen concentrations on imprinted methylation. Here, we compared the effects of SO+EC using low and high O_2_ concentrations [5% O_2_ and ~ 21% O_2_ in ambient air, respectively] on imprinted methylation maintenance. No significant difference was found in the frequency of embryos with *Snrpn*, *Kcnq1ot1*, and *H19* methylation loss in SO+EC groups whether cultured in 5% or ~ 21% O_2_ (Fig. 4; Additional file 1: Figures S6, S7, and S8; Additional file 1: Table S1). These results are consistent with those reported by de Waal et al. [56] and Ghosh et al. [57], who reported no difference in DNA methylation perturbations in mouse and human placentas between the two oxygen tensions, although both differed from controls.

For all treatment comparisons, no significant change in methylation of the normally unmethylated allele was found for any gene. Overall, these results indicate that the frequency of imprinted methylation perturbations was similar between SO and EC, while for ICRs harboring maternal methylation, SO+EC produces an additive effect with a greater frequency of blastocysts with perturbations in imprinted methylation than SO alone.

One question that arises from these experiments is whether all females undergoing ARTs produce embryos with imprinted methylation errors, or whether some females have embryos that maintain imprinted methylation while other females generate embryos that lose imprinted methylation. To address this question, blastocysts that were analyzed above according to ART group were also evaluated by individual litters within a treatment group. Compared to controls, every litter had at least one blastocyst that lost imprinted methylation in the SO only, EC only, and SO+EC groups with the frequency of imprinted methylation errors reflective of the conditions (Additional file 1: Figure S9). These data indicate that it is exclusively ARTs acting at the individual embryo level, rather than a female or batch effect, which contributes to imprinted methylation errors.

### No additional burden of imprinted methylation maintenance errors with a combination of advanced maternal age and ARTs

**Fig. 5:**
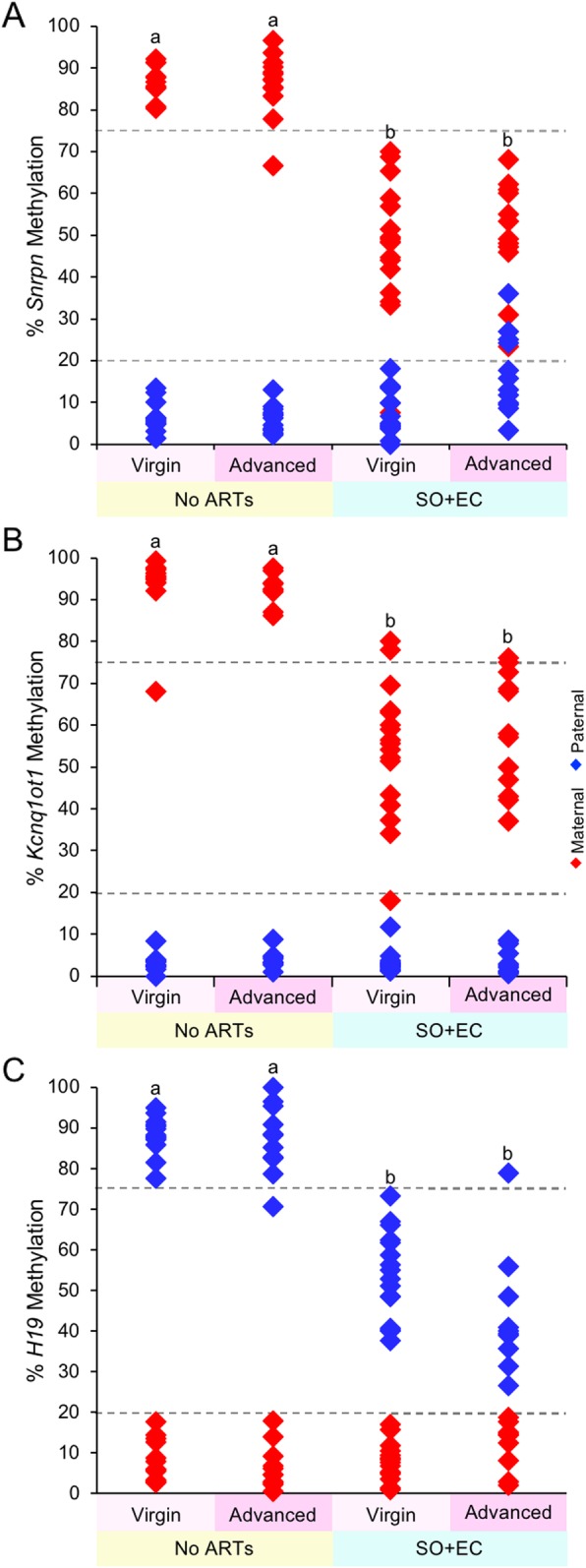
Increasing maternal age plus ARTs did not further increase imprinted methylation errors at the (**a**) *Snrpn*, (**b**) *Kcnq1ot1*, and (**c**) *H19* ICRs in blastocysts. Diamonds represent the mean methylation levels of maternal (red) or paternal (blue) alleles for *Snrpn*, *Kcnq1ot1*, and *H19* ICRs in individual embryos (*n* = 10–17 blastocysts; *n* = 2–5 females per treatment group). Letters (a, b, c), a statistically significant difference between groups (Additional file 1: Table S2)

Finally, we tested the hypothesis that a combination of advanced maternal age and ARTs would result in a higher frequency of blastocysts with imprinted methylation errors than ARTs alone. To test this hypothesis, we compared embryos in the virgin and advanced age no ARTs groups with those in the virgin and advanced age SO+EC groups. A significant loss in imprinted methylation occurred in blastocysts from the advanced age SO+EC group compared to advanced age no ARTs group, similar to these groups from virgin females (Fig. 5; Additional file 1: Figures S10, S11, and S12; Additional file 1: Table S2). However, the comparison between virgin SO+EC and advanced maternal age plus SO+EC revealed no significant change in the frequency of blastocysts with imprinted methylation perturbations at the maternal *Snrpn*, maternal *Kcnq1ot1*, and paternal *H19* ICRs. These data indicate that advanced maternal age does not further increase the burden of imprinted methylation errors when combined with ARTs.

## Discussion

As women increasingly delay childbirth, it is important to understand the effects of reproductive aging on oocyte and embryo quality so that women can be informed of the potential risks to both the pregnancy and the future health of the baby. As fertility declines with increasing maternal age, many women turn to fertility treatments to conceive [3], further contributing to the environmental stressors on the embryo. However, to date, there has been little investigation on the effects of advanced maternal age, with or without ARTs, on genomic imprinting. Overall, our data showed that while ARTs alter imprinted methylation maintenance of *Snrpn*, *Kcnq1ot1*, and *H19* in preimplantation embryos, advanced maternal age had no effect on imprinted methylation acquisition in oocytes or on imprinted methylation maintenance in blastocysts. Moreover, advanced maternal age did not further increase the burden of imprinted methylation errors at *Snrpn*, *Kcnq1ot1*, and *H19* when combined with ARTs. These results support cautious optimism that advanced maternal age is not a contributing factor to imprinted methylation errors in embryos produced in the clinic. Furthermore, our data on the effects of ARTs strengthen the need to advance clinical methods to reduce imprinted methylation errors in in vitro-produced embryos.

The results of our study are consistent with those reported by others. A comparison of young (2–2.5 months) and advanced aged (~ 10–11 months) females failed to detect any change in imprinted methylation in E10.5 embryos, although there was greater embryo demise [58]. Our analysis of imprinted methylation in blastocysts from females of increasing age suggests that this embryo wastage was not due to imprinted methylation maintenance errors arising during preimplantation development. In another study, DNA methylation was unchanged at imprinted genes in E16.5 embryos from advanced aged (15 months old) females compared to young (~ 1 month old) mothers, although an increase in DNA methylation at imprinted genes was reported for E16.5 placentas from advanced aged mothers (15 months old) [59]. It should be noted only 1–2 CpG sites were assessed in this analysis for total methylation, so it is unclear which parental allele may have gained methylation. Finally, in another report that investigated the epidemiological association in Japan between imprinting disorders, ARTs and advanced maternal age found that ART-conceived children with PWS were more likely to have *Snrpn* methylation errors than spontaneously conceived children with PWS when their mothers were less than 37 years old [35]. However, when mothers were more than 38 years old, there was no difference in the frequency of PWS caused by DNA methylation errors in spontaneously versus ART-conceived children. Instead, a significantly higher risk of maternal uniparental disomy occurred in ART-conceived children of these mothers, suggesting that the etiology of imprinting disorders in children of women of advanced maternal age was due to chromosomal segregation errors in aged oocytes. Thus, the effect of ARTs on imprinted DNA methylation was independent of maternal age, which supports our conclusions. Overall, the evidence that advanced maternal age did not further increase the burden of imprinted methylation errors when combined with ARTs provides important information for women over the age of 35.

Numerous ARTs are employed for in vitro production of mouse and human embryos. A complete clinical cycle may include ovarian stimulation, oocyte retrieval, in vitro fertilization or intracytoplasmic injection, embryo culture, oocyte or embryo cryopreservation, and embryo transfer to mothers. Several studies on the effects of multiple ARTs reported that combining ARTs increased the chance of imprinted methylation and expression errors when compared to single interventions [39, 40, 60–62]. Here, we found that SO+EC produced an additive effect with a greater frequency of blastocysts with perturbations in maternal *Snrpn* and *Kcnq1ot1* imprinted methylation than SO alone. These results suggest that SO and EC have independent effects on genes harboring maternal methylation. In contrast, there was no significant difference in the frequency of blastocysts with *H19* imprinted methylation perturbations between the SO only, EC only, and SO+EC groups. This may relate to the fact that *H19* possesses paternal, sperm-inherited methylation, and suggests that SO and EC also converge on the same molecular pathway during preimplantation development. Future studies are needed to investigate the molecular mechanisms regulating imprinted methylation maintenance and how they may be perturbed by ARTs.

While the focus of this study was centered on imprinted methylation, the possibility exists that advanced maternal age may impair DNA methylation or histone modifications at other regions of the genome. In one study, global methylation that was assessed by immunofluorescence with 5-mC antibody showed a slight but significant decrease in signal in preimplantation embryos from middle-aged mice (8–10 months) compared to young females (1.5–2 months) [14]. Three epigenome-wide association studies examining global DNA methylation in children of older mothers found individual CpG sites within different genomic regions that were significantly correlated with advanced maternal age [63–65]. However, Ghosh et al. [57] reported that while global and LINE1 methylation errors differed between control and ART placentas of young mothers (< 35 years old), there was little difference in DNA methylation in older mothers (> 35 years old), pointing towards ARTs rather than maternal age as a contributing factor. In three studies that investigated histone modifications, histone 4 lysine 12 (H4K12) acetylation was improperly retained in MII oocytes from older mice and humans [66, 67], while histone methylation (H3K9me3, H3K36me2, H3K79me2, and H4K20me2) was abnormally decreased in GV and MII oocytes from advanced age (11 months old) compared to young (2 months old) mice [68]. Thus, future investigations are required to better understand the effects of advanced maternal age, with and without ARTs, on global DNA methylation and histone modifications.

A limitation of this study is the longevity of our animal model as compared to that of humans. While we found that female mice of advanced maternal age experienced a decreased ovarian reserve, producing fewer oocytes and embryos, the actual time of reproduction was maximally 14 months in our model. For humans, as a woman ages, so do her oocytes, with some oocytes reaching over 40 years before growth is initiated. The absolute time period for reproductive aging of 35–55 years could have significant impact on the ability to acquire and maintain imprinted methylation. Having said this, in our previous study on donated human preimplantation embryos, we found that the mean maternal age for embryos with normal methylation and abnormal levels at imprinted genes was 34 and 33 years old, which was not statistically different [42]. This suggests that advanced maternal age may not be an additional burden for imprinted methylation maintenance in humans, similar to the findings reported here for mice. To confirm this, future studies on donated human oocytes and preimplantation embryos from advanced aged women are needed to determine the impact of decades-long oocyte aging.

## Conclusion

Increasing maternal age with or without superovulation had no effect of imprinted methylation acquisition in oocytes. While ARTs generated perturbations in imprinted methylation maintenance in blastocysts, advanced maternal age did not increase the burden of imprinted methylation errors when combined with ARTs. Overall, these results indicate that ARTs, and not maternal age, are the major contributor to imprinted methylation errors. Going forward, it is crucial to ascertain the molecular mechanisms underlying these imprinted methylation perturbations, as well as develop minimally invasive markers for identifying embryos with altered imprinted methylation. Knowledge gained from these studies will lead to advances that reduce the frequency of embryos with imprinting perturbations, and ultimately, ART-conceived children with imprinting disorders.

## Methods

### Mice

In this study, we utilized a mouse model suited for imprinting analyses, namely C57BL/6(CAST7) [B6(CAST7)] mice [69]. B6(CAST7) females had two copies of the *Mus musculus castaneus* chromosome 7 (CAST7) on a C57BL/6(B6) background, while B6 males (Jackson Laboratory) had two copies of the B6 chromosome 7; therefore, the offspring of these mice have one CAST7 chromosome 7 from the mother and one B6 chromosome 7 from the father. The mouse chromosome 7 contains numerous imprinted genes, including those investigated in this study: *Snrpn*, *Kcnq1ot1*, and *H19*. Multiple polymorphisms between the CAST7 and B6 alleles allow for the distinction of maternal versus paternal alleles, respectively, at imprinted loci on chromosome 7 in an embryo.

### Maternal aging model

We produced a model for maternal aging, extending from puberty to reproductive senescence, that more precisely relates human and mouse ages [44, 45]. Female mice were then divided into four age groups (Additional file 1: Figure S1): virgin females between 1.5 to 2 months old that served as controls, young maternal age between > 2–6 months old, middle maternal age between > 6–10 months old, and advanced maternal age between > 10–14 months of age. To control for possible subfertility, retired female breeder mice were used for the young, middle, and advanced age groups. These females at 2 months of age were set up with males as an active breeding pair and fertility was measured by the ability to continuously produce litters. Once they reach the targeted maternal ages, the breeding pair was dismantled, and the female was designated as a retired breeder with proven fertility. Each female was weighed to distill any effects of weight versus age. B6 male studs between 2 and 6 months of age were mated with control and experimental females. Matings were determined by the presence of a vaginal plug at 0.5 days post-coitum.

### Assisted reproductive technologies

There were four treatment groups in this study: no ARTs, superovulation only, embryo culture only, and superovulation plus embryo culture. For the no ARTs group, germinal vesicle (GV) oocytes and blastocysts were collected from spontaneously ovulating females on embryonic day 3.5 (E3.5) in M2 media (Sigma). For GV oocyte isolation, ovaries were incubated in collagenase in M2 media (2 mg/mL; Sigma) for 15 min at 37 °C [51]. Follicles were collected and incubated in 0.05% trypsin/EDTA in PBS (Sigma) for 15 min at 37 °C to remove cumulus cells. Our group and others [51, 70] have demonstrated that acquisition of methylation marks at imprinted genes, including *Snrpn*, was completed by the late secondary follicle or early GV stages, by the time the oocyte is 60 μm in diameter. For this reason, oocytes that were smaller than 60 μm in diameter were excluded, with the majority of the oocytes analyzed falling between 70–80 μm in diameter. To further prevent cumulus cell contamination, zonae pellucidae were removed from all oocytes by incubating the oocytes in 100% acidic Tyrode’s solution (Sigma) for < 1 min at room temperature.

For the superovulation only group, females received intraperitoneal (IP) injections of 10 IU equine chorionic gonadotropin (eCG; Sigma) followed by 10 IU human chorionic gonadotropin (hCG; Sigma) 46 h later [40]. Blastocysts were collected as described above. Superovulated MII oocytes were collected from the oviducts 18 h after the hCG injection and treated with 0.3 mg/ml hyaluronidase in M2 medium (0.3 mg/mL; Sigma) to remove cumulus cells. Zonae pellucidae were removed from all MII oocytes as described above.

For the embryo culture only group, two-cell embryos were collected from the oviducts at E1.5 in M2 medium and washed in Whitten’s medium [71]. Embryos were cultured for 3 days in Whitten’s medium covered in mineral oil at 5% CO_2_, 5% O_2_, and 37 °C until the blastocyst stage.

For the superovulation and embryo culture group, females were superovulated as above, mated with B6 males, and two-cell embryos were collected on E1.5 and cultured for 3 days in Whitten’s medium [71] covered in mineral oil at either 5% CO_2_, 5% O_2_, and 90% N_2,_ or 5% CO_2_ in air (21% O_2_) at 37 °C until the blastocyst stage. Note that in these experiments, we used 10 IU hormone dosages and/or embryo culture in Whitten’s medium as the most tractable system for investigating imprinted methylation errors [39, 40].

### Imprinted DNA methylation analysis of individual oocytes and blastocysts

All samples were processed immediately after collection without freezing. Individual oocytes or embryos were embedded into 10 μl 2:1 agarose to lysis solution beads (20 μL 3% low melting point agarose; 8 μL lysis buffer [100 mM Tris–HCl, pH 7.5 (Bioshop), 500 mM LiCl (Sigma), 10 mM ethylenediaminetetraacetic acid, pH 8.0 (Sigma), 1% LiDS (Bioshop), and 5 mM 1,4-dithiothreitol (Sigma)], 1 μl 2 mg/mL proteinase K (Sigma), and 1 μl 0.05% Igepal (Sigma) under mineral oil [72]. A negative control (agarose bead without oocyte or embryo) was processed alongside for every eight samples. Bisulfite mutagenesis, nested PCR, and clonal sequencing were performed as described previously [72]. Briefly, samples in agarose beads were incubated in SDS lysis buffer (1% sodium dodecyl sulfate in Tris-EDTA) at 50 °C overnight. The following day, the lysis buffer was replaced with mineral oil and samples were incubated at 90 °C for 2.5 min to inactive the proteinase K, then placed on ice for 10 min. The DNA was denatured by incubation in 0.1 M sodium hydroxide at 37 °C for 15 min. Samples were incubated in a 2.5 M sodium bisulfite solution (3.8 g sodium bisulfite, 1 mL 0.125 M hydroquinone, and 1 mL 3 M sodium hydroxide in 5.5 mL water) under mineral oil at 50 °C for 3–3.5 h depending on target gene (Additional file 1: Table S3). Samples were desulfonated with 0.3 M sodium hydroxide at 37 °C for 15 min, and then were washed twice in Tris-EDTA and twice in water for 6 min each with shaking. Following sodium bisulfite treatment, DNA was amplified via two rounds of nested PCR using primers previous described for *Snrpn*, *Kcnq1ot1*, or *H19* [40] (Additional file 1: Table S3). Agarose beads were added to the first round of PCR reactions containing Hot Start Ready-to-Go PCR beads (GE Healthcare), containing 0.2 μM final concentration of outer primers and 9.6 ng/ml final concentration of tRNA as a carrier in the first-round reaction with mineral oil overlay. For the second-round reaction, 5 μl of first round was added to a second 25 μl ready-to-go PCR bead containing 0.2 μM final concentration of the inner primers with mineral oil overlay. See Additional file 1: Table S3 for primers. Following successful amplification, second round PCR products were ligated with the pGEMT-EASY DNA ligation kit (Promega) overnight at 4 °C. Ligations were transformed into competent DH5α *Escherichia coli* cells (Zymo Research). Cloning success was determined with blue/white selection on LB/ampicillin/isopropyl β-D-1-thiogalactopyranoside/x-galactose agar plates. For clonal sequencing, individual bacterial colonies were subjected to colony PCR with M13 primers (forward 5′ to 3′ CGCCAGGGTTTTCCCAGTCACGAC; reverse 5′ to 3′ TCACACAGGAAACAGCTATGAC). PCR amplicons of the correct size for each gene were verified by gel electrophoresis and were sent to Bio-Basic Inc. (Markham, QC, Canada) for sequencing. For oocytes, 5–8 clones were sequenced. Oocytes were excluded if the clones had more than one methylation pattern, suggestive of cumulus cell contamination. For embryos, 25–50 clones were sequenced from each sample for a final target of at least 7 independent clones each of maternal and paternal origin. Sequences were excluded for clones with less than 85% conversion; CAST7 and B6 polymorphisms in the same sequence, indicative of crossover; and identical number and location of unconverted CpG and non-CpG associated cytosines. Embryos were excluded if clones were of only maternal or paternal origin, indicating biased amplification. The methylation percentages for all unique maternal or paternal allele clones in an embryo were averaged to obtain the average methylation on the maternal or paternal alleles for the embryo.

### Statistical analysis

Fisher’s exact test (https://www.langsrud.com/fisher.htm) was used to calculate the significance of non-random association between groups of embryos. For the methylated maternal *Snrpn*, maternal *Kcnq1ot1*, or paternal *H19* allele, embryos with > 75% methylation were designated as maintaining methylation and embryos with ≤ 75% methylation were designated as losing methylation. For the unmethylated paternal *Snrpn*, paternal *Kcnq1ot1*, or maternal *H19* allele, embryos with ≤ 20% methylation maintained methylation and embryos with > 20% methylation gained methylation. A one-sided, right-tailed test was used, as methylation changes were expected to be only in one direction (increase on the unmethylated allele or decrease on the methylated allele). *p* values were considered significant at *p* < 0.05.

## Additional file

**Additional file 1: Figure S1. Correlation of mouse and human ages on which we based our mouse maternal age model. Figure S2. Imprinted methylation acquisition at the normally methylated maternal** ***Snrpn*** **ICR in individual mouse blastocysts from mothers of increasing age.** Embryos from spontaneously ovulated **(A)** virgin (1.5 to 2 months old), **(B)** young maternal age (>2-6 months old), **(C)** middle maternal age (>6-10 months old), and **(D)** advanced maternal age (>10 months old) females (n=10-22 embryos; n=3-7 females per age group). Each box encloses embryos analyzed from one female. Each block represents an individual embryo. Each line denotes an individual strand of DNA with the maternal allele [C57BL/6(CAST7)] on the left and the paternal allele (C57BL/6) on the right. Embryo designation is at the top left of each block and percent methylation is at the top right. Black circles, methylated CpGs; white circles, unmethylated CpGs; ARTs, assisted reproductive technologies. **Figure S3. Imprinted methylation acquisition at the normally methylated maternal** ***Kcnq1ot1*** **ICR in individual blastocysts from mothers of increasing age.** Embryos from spontaneously ovulated **(A)** virgin (1.5 to 2 months old), **(B)** young maternal age (>2-6 months old), **(C)** middle maternal age (>6-10 months old), and **(D)** advanced maternal age (>10 months old) females (n=9-16 embryos; n=3-5 females per age group). See Figure S2 for details. **Figure S4. Imprinted methylation acquisition at the normally methylated paternal** ***H19*** **ICR in individual blastocysts from mothers of increasing age.** Embryos from spontaneously ovulated **(A)** virgin (1.5 to 2 months old), **(B)** young maternal age (>2-6 months old), **(C)** middle maternal age (>6-10 months old), and **(D)** advanced maternal age (>10 months old) females (n=11-21 embryos; n=3-6 females per age group). See Figure S2 for details. **Figure S5. Imprinted methylation maintenance unaltered by maternal weight. (A)** Positive correlation between maternal age and maternal weight. Black circles indicate female mice from which both oocytes and blastocysts were collected; white circles indicate female mice from which only oocytes were collected. **(B-D)** No association between maternal weight and imprinted methylation at the **(B)** *Snrpn,* **(C)** *Kcnq1ot1*, or **(D)** *H19* ICRs. Diamonds represent the mean methylation of maternal (red) or paternal (blue) alleles for individual embryos. **Figure S6. Imprinted methylation maintenance at the normally methylated maternal** ***Snrpn*** **ICR in individual blastocysts from virgin females after ART treatment. (A)** SO only; **(B)** EC only with 5% O_2_; **(C)** SO+EC with 5% O_2_; **(D)** SO+EC with 21% O_2_ (n=10-13 embryos; n=3 females per treatment group). Each box encloses embryos analyzed from one female. Each block denotes an individual embryo. Each line denotes an individual strand of DNA with the maternal allele [C57BL/6(CAST7)] on the left and the paternal allele (C57BL/6) on the right. Embryo designation is at the top left of each block and percent methylation is at the top right. Black circles indicate methylated CpGs, white circles indicate unmethylated CpGs. SO, superovulation; EC, *in vitro* embryo culture. **Figure S7. Imprinted methylation maintenance at the normally methylated maternal** ***Kcnq1ot1*** **ICR in individual blastocysts from virgin females after ART treatment. (A)** SO only; **(B)** EC only with 5% O_2_; **(C)** SO+EC with 5% O^2^; **(D)** SO+EC with 21% O_2_ (n=10-17 embryos; n=3-4 females per treatment group). See Figure S6 for details. **Figure S8. Imprinted methylation maintenance at the normally methylated paternal** ***H19*** **ICR in individual blastocysts from virgin females after ART treatment. (A)** SO only; **(B)** EC only with 5% O_2_; **(C)** SO+EC with 5% O_2_; **(D)** SO+EC with 21% O_2_ (n=9-16 embryos; n=3 females per treatment group). See Figure S6 for details. **Figure S9. At least one blastocyst from every ART-treated litter lost imprinted methylation on the normally methylated** **(A)** ***Snrpn,*** ***(B) Kcnq1ot1,*** **or (C)** ***H19*** **ICRs.** Diamonds represent the average percent methylation of maternal (red) or paternal (blue) alleles for individual embryos. ARTs, assisted reproductive technologies; SO and S, superovulation; EC and C, embryo culture; SC, superovulation plus embryo culture. **Figure S10. Imprinted methylation maintenance at the normally methylated maternal** ***Snrpn*** **ICR in individual blastocysts from advanced maternal age females after ART treatment.** Each box encloses embryos analyzed from one female. Each block denotes an individual embryo. Each line represents an individual strand of DNA with the maternal allele [C57BL/6(CAST7)] on the left and the paternal allele (C57BL/6) on the right. Embryo designation is at the top left of each block and percent methylation is at the top right. Black circles, methylated CpGs; white circles, unmethylated CpGs; SC, superovulation plus embryo culture. **Figure S11. Imprinted methylation maintenance at the normally methylated maternal** ***Kcnq1ot1*** **ICR in individual blastocysts from advanced maternal age females after ART treatment.** See Figure S10 for details. **Figure S12. Imprinted methylation maintenance at the normally methylated paternal** ***H19*** **ICR in individual blastocysts from advanced maternal age females after ART treatment.** See Figure S10 for details. **Table S1. Comparison of treatment groups using Fisher exact test. Table S2. Comparison of maternal age and treatment groups using Fisher exact test. Table S3. Bisulfite mutagenesis and PCR amplification.**

## Data Availability

The datasets supporting the conclusions of this article are included within the article and its additional file.

## Abbreviations

ART: Assisted reproductive technology
AS: Angelman syndrome
BWS: Beckwith-Wiedemann syndrome
DNMT: DNA methyltransferase
EC: Embryo culture
eCG: Equine chorionic gonadotropin
GV: Germinal vesicle
hCG: Human chorionic gonadotropin
ICR: Imprinting control region
MII: Meiosis II
PWS: Prader-Willi syndrome
SO: Superovulation
SRS: Silver-Russell syndrome
