# Supplementary material for: Perturbations in imprinted methylation from assisted reproductive technologies but not advanced maternal age in mouse preimplantation embryos
Source: Clin Epigenetics. 2019 Nov 26;11:162. doi: 10.1186/s13148-019-0751-9 (PMC6878706; doi:10.1186/s13148-019-0751-9)
Supplement: Supplementary file 1 — Additional file 1: Figure S1. Correlation of mouse and human ages on which we based our mouse maternal age model. Figure S2. Imprinted methylation acquisition at the normally methylated maternal Snrpn ICR in individual mouse blastocysts from mothers of increasing age. Embryos from spontaneously ovulated (A) virgin (1.5 to 2 months old), (B) young maternal age (>2-6 months old), (C) middle maternal age (>6-10 months old), and (D) advanced maternal age (>10 months old) females (n=10-22 embryos; n=3-7 females per age group). Each box encloses embryos analyzed from one female. Each block represents an individual embryo. Each line denotes an individual strand of DNA with the maternal allele [C57BL/6(CAST7)] on the left and the paternal allele (C57BL/6) on the right. Embryo designation is at the top left of each block and percent methylation is at the top right. Black circles, methylated CpGs; white circles, unmethylated CpGs; ARTs, assisted reproductive technologies. Figure S3. Imprinted methylation acquisition at the normally methylated maternal Kcnq1ot1 ICR in individual blastocysts from mothers of increasing age. Embryos from spontaneously ovulated (A) virgin (1.5 to 2 months old), (B) young maternal age (>2-6 months old), (C) middle maternal age (>6-10 months old), and (D) advanced maternal age (>10 months old) females (n=9-16 embryos; n=3-5 females per age group). See Figure S2 for details. Figure S4. Imprinted methylation acquisition at the normally methylated paternal H19 ICR in individual blastocysts from mothers of increasing age. Embryos from spontaneously ovulated (A) virgin (1.5 to 2 months old), (B) young maternal age (>2-6 months old), (C) middle maternal age (>6-10 months old), and (D) advanced maternal age (>10 months old) females (n=11-21 embryos; n=3-6 females per age group). See Figure S2 for details. Figure S5. Imprinted methylation maintenance unaltered by maternal weight. (A) Positive correlation between maternal age and maternal weight. Black circl [file 13148_2019_751_MOESM1_ESM.pdf]

Perturbations in imprinted methylation from assisted reproductive technologies but not advanced maternal age in mouse preimplantation embryos

Audrey J. Kindsfather[1,2], Megan A. Czekalski[1,2], Catherine A. Pressimone[1,2], Margaret P. Erisman[1,2] and Mellissa R.W. Mann[1,2*]

[1] Department of Obstetrics, Gynaecology and Reproductive Sciences, University of Pittsburgh School of Medicine, Pittsburgh, Pennsylvania, USA
[2] Magee-Womens Research Institute, Pittsburgh, Pennsylvania, 15213 USA

*Correspondence: mannmr@mwri.magee.edu

Additional Files

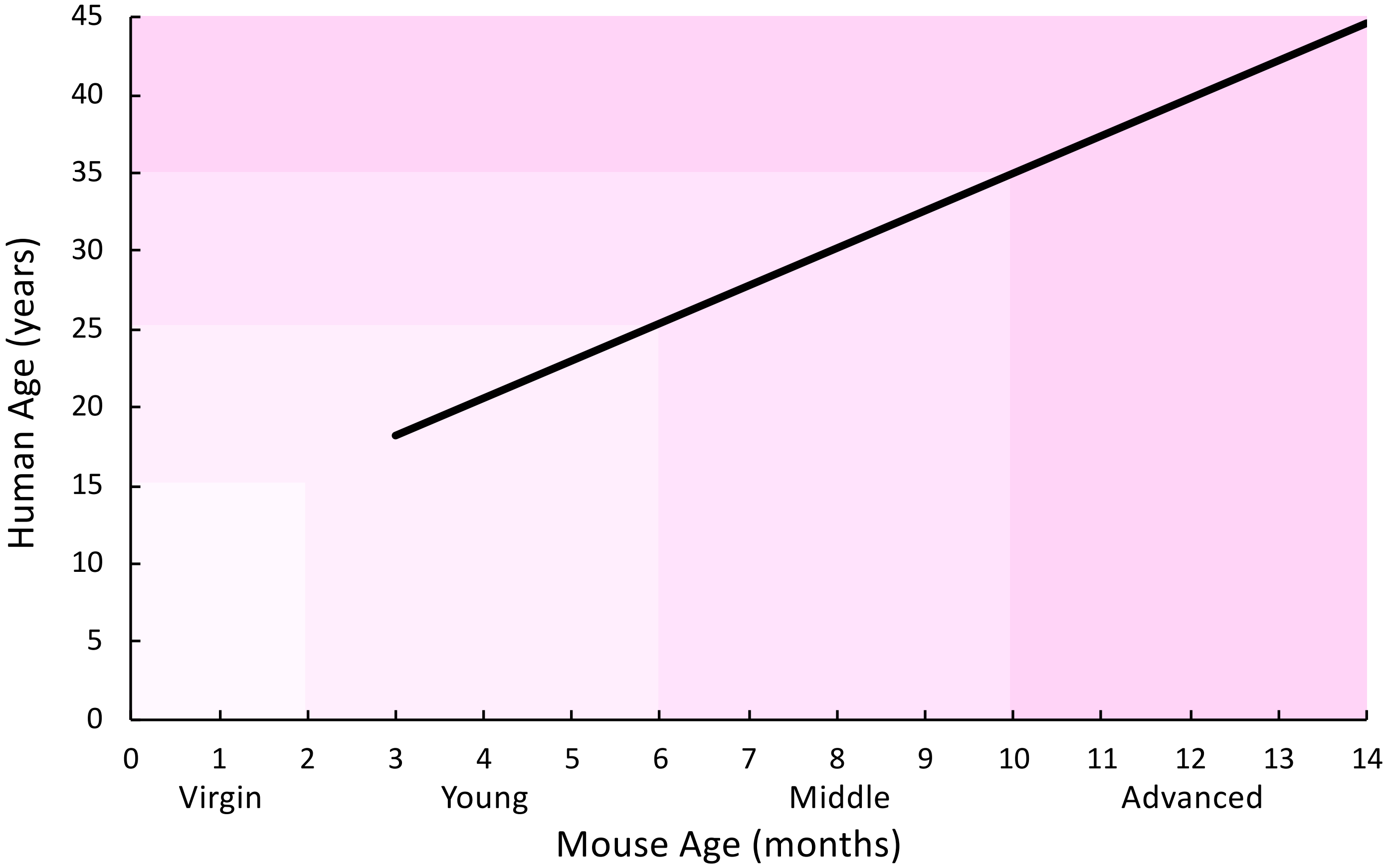

Figure S1. Correlation of mouse and human ages on which we based our mouse maternal age model.

Kindsfather Figure S2

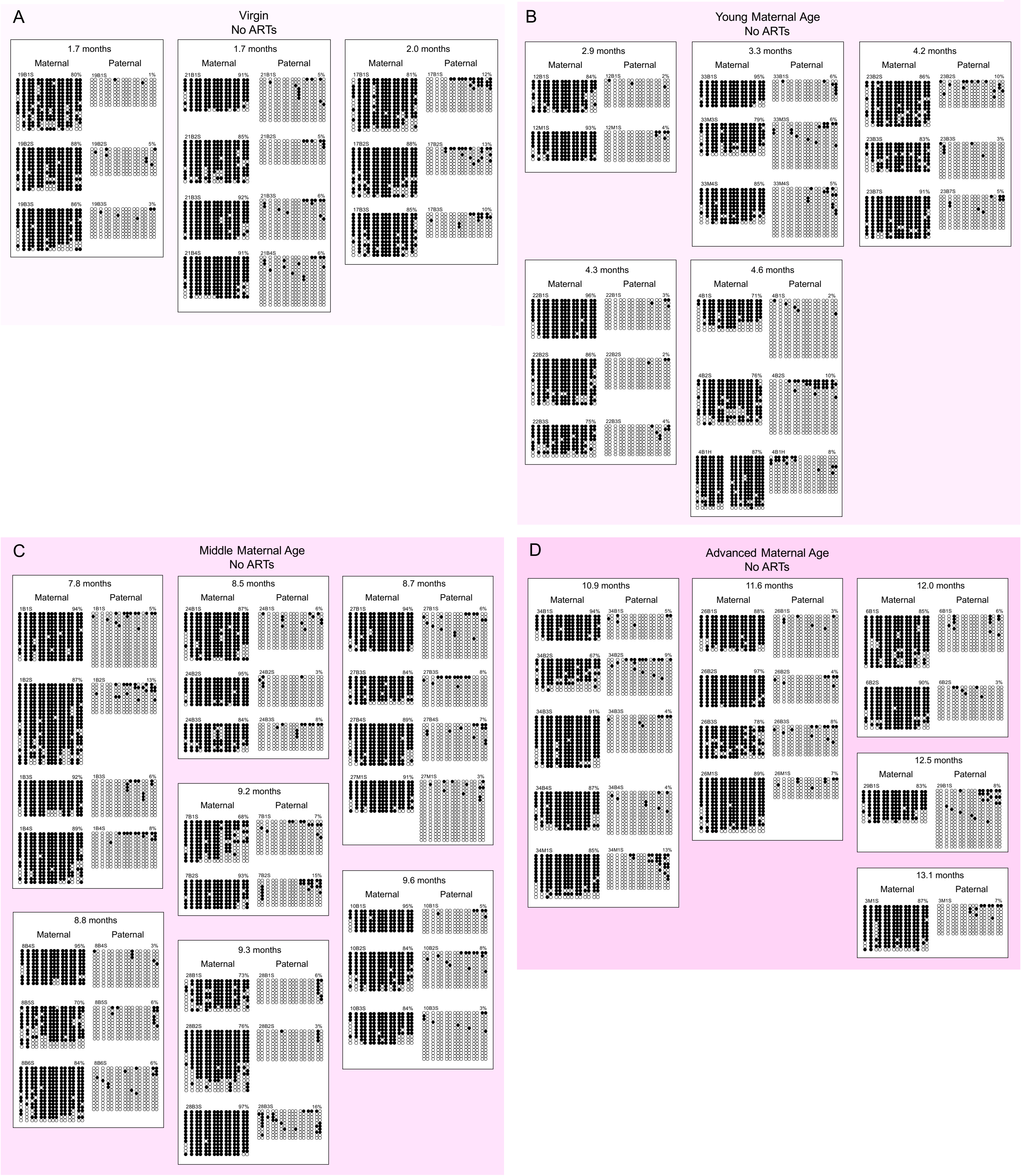

Figure S2. Imprinted methylation acquisition at the normally methylated maternal *Snrpn* ICR in individual mouse blastocysts from mothers of increasing age. Embryos from spontaneously ovulated **(A)** virgin (1.5 to 2 months old), **(B)** young maternal age (>2-6 months old), **(C)** middle maternal age (>6-10 months old), and **(D)** advanced maternal age (>10 months old) females (n=10-22 embryos; n=3-7 females per age group). Each box encloses embryos analyzed from one female. Each block represents an individual embryo. Each line denotes an individual strand of DNA with the maternal allele [C57BL/6(CAST7)] on the left and the paternal allele (C57BL/6) on the right. Embryo designation is at the top left of each block and percent methylation is at the top right. Black circles, methylated CpGs; white circles, unmethylated CpGs; ARTs, assisted reproductive technologies.

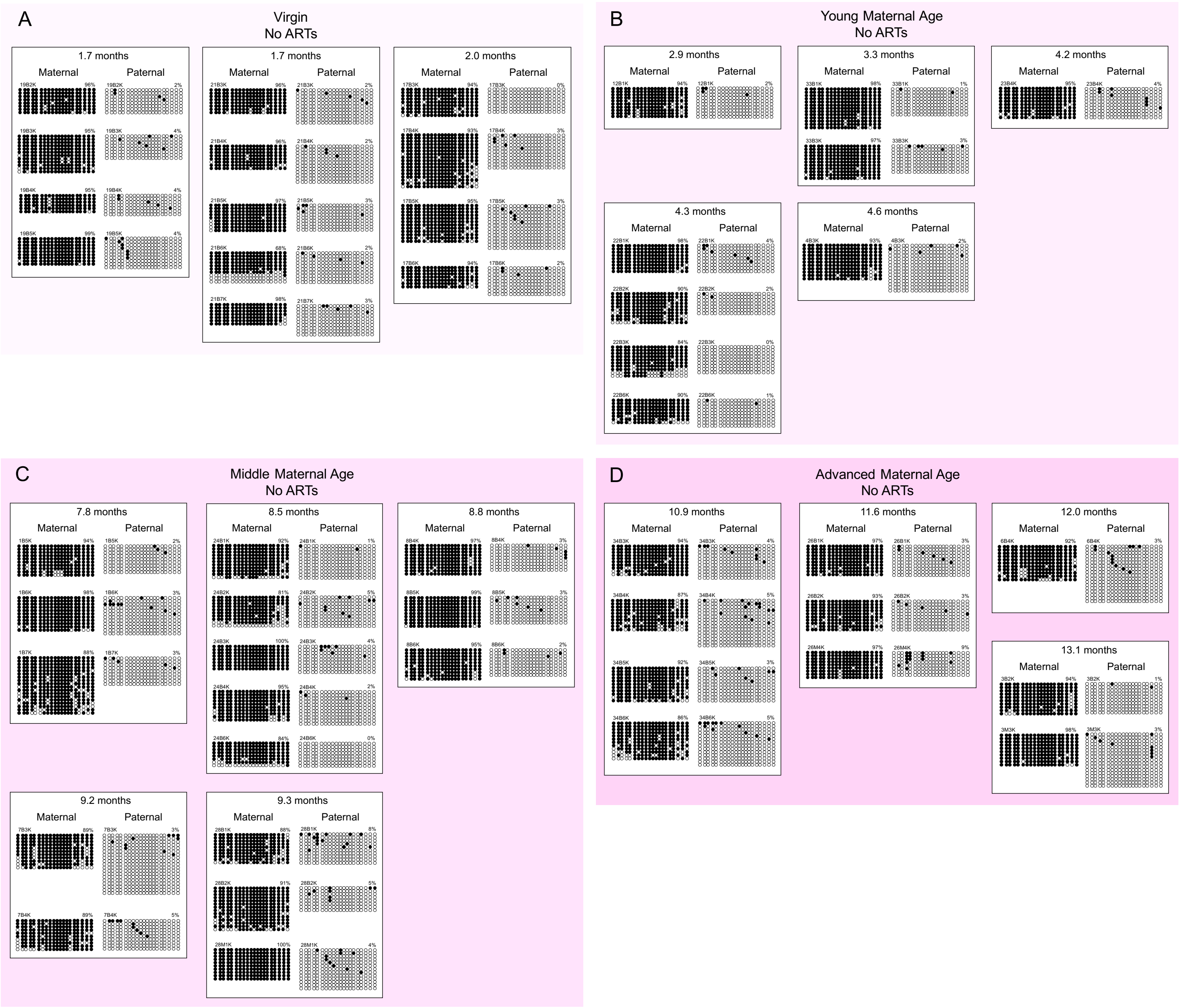

Figure S3. Imprinted methylation acquisition at the normally methylated maternal *Kcnq1ot1* ICR in individual blastocysts from mothers of increasing age. Embryos from spontaneously ovulated **(A)** virgin (1.5 to 2 months old), **(B)** young maternal age (>2-6 months old), **(C)** middle maternal age (>6-10 months old), and **(D)** advanced maternal age (>10 months old) females (n=9-16 embryos; n=3-5 females per age group). See Figure S2 for details.

Kindsfather Figure S4

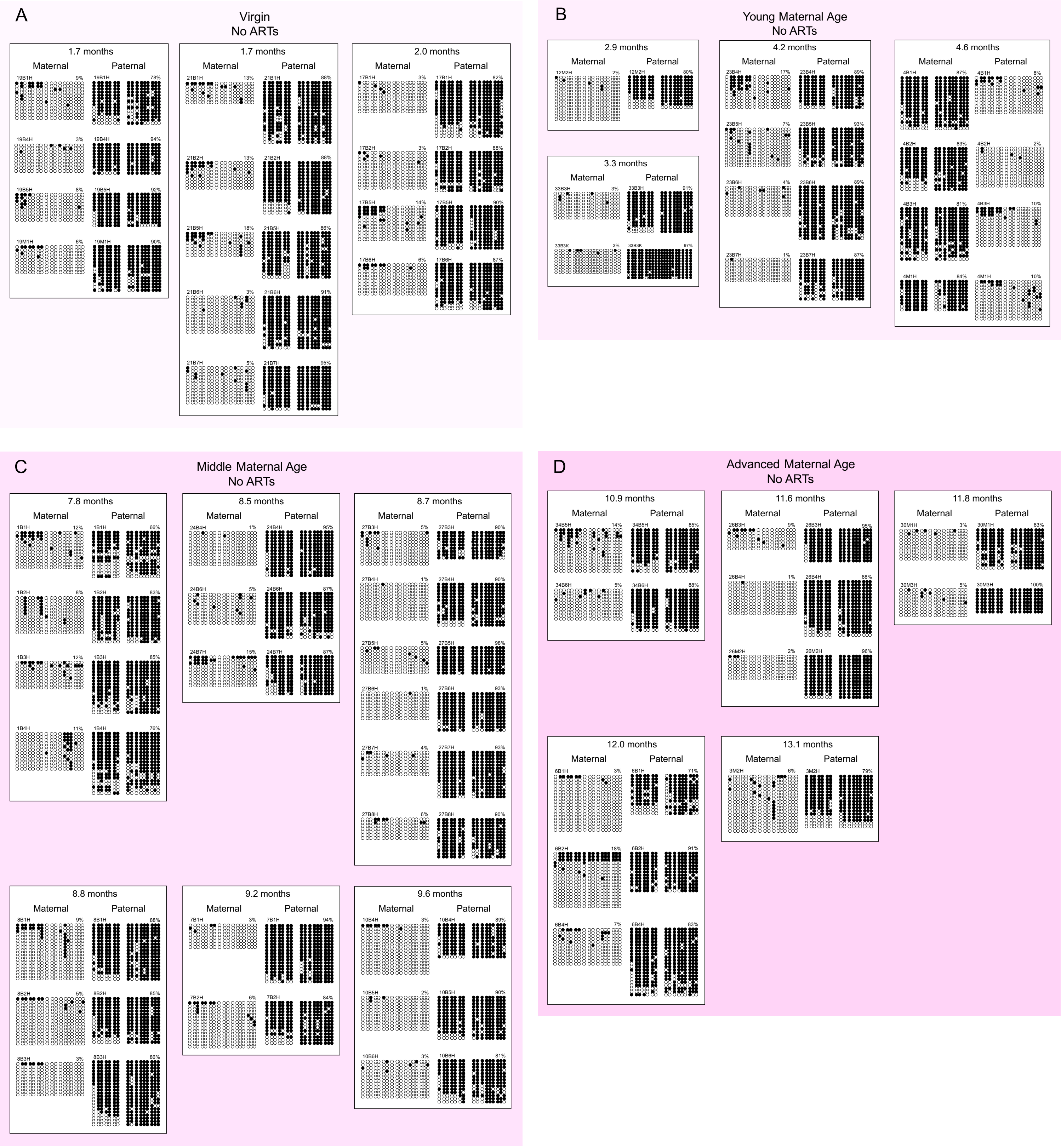

Figure S4. Imprinted methylation acquisition at the normally methylated paternal *H19* ICR in individual blastocysts from mothers of increasing age. Embryos from spontaneously ovulated **(A)** virgin (1.5 to 2 months old), **(B)** young maternal age (>2-6 months old), **(C)** middle maternal age (>6-10 months old), and **(D)** advanced maternal age (>10 months old) females (n=11-21 embryos; n=3-6 females per age group). See Figure S2 for details.

Kindsfather Figure S5

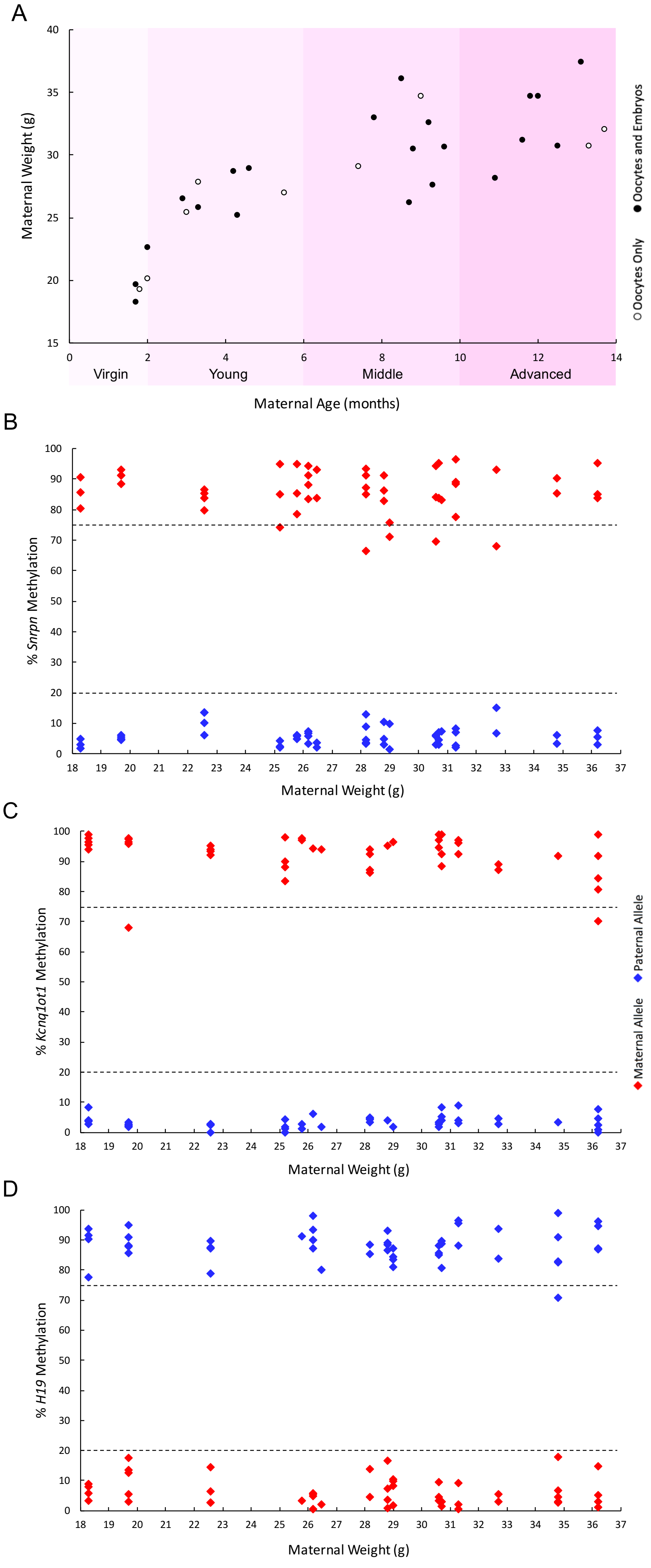

Figure S5. **Imprinted methylation maintenance unaltered by maternal weight. (A)** Positive correlation between maternal age and maternal weight. Black circles indicate female mice from which both oocytes and blastocysts were collected; white circles indicate female mice from which only oocytes were collected. **(B-D)** No association between maternal weight and imprinted methylation at the **(B)** *Snrpn,* **(C)** *Kcnq1ot1*, or **(D)** *H19* ICRs. Diamonds represent the mean methylation of maternal (red) or paternal (blue) alleles for individual embryos.

Kindsfather Figure S6

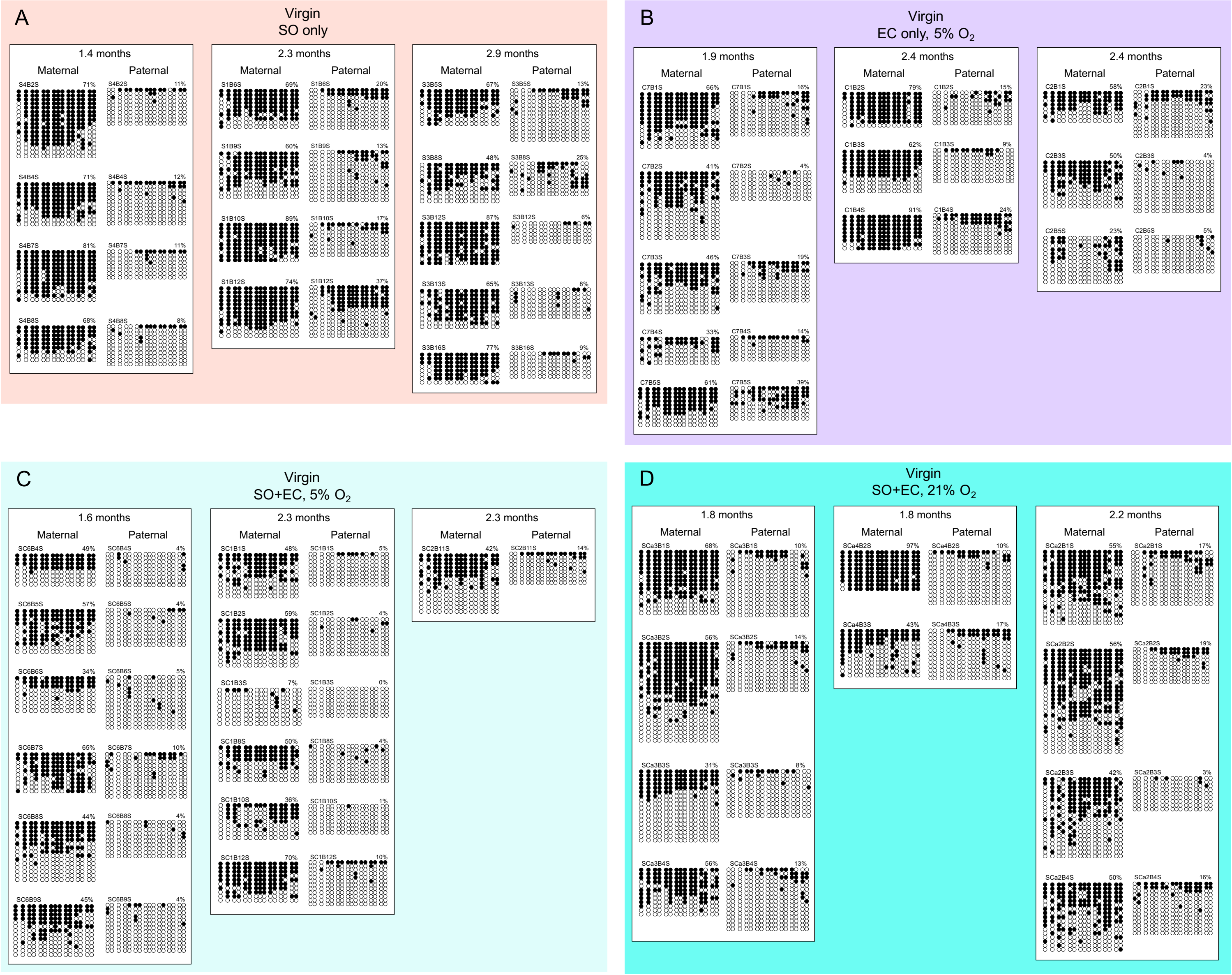

Figure S6. Imprinted methylation maintenance at the normally methylated maternal *Snrpn* ICR in individual blastocysts from virgin females after ART treatment. (A) SO only; **(B)** EC only with 5% O_2; **(C)** SO+EC with 5% O_2; **(D)** SO+EC with 21% O_2 (n=10-13 embryos; n=3 females per treatment group). Each box encloses embryos analyzed from one female. Each block denotes an individual embryo. Each line denotes an individual strand of DNA with the maternal allele [C57BL/6(CAST7)] on the left and the paternal allele (C57BL/6) on the right. Embryo designation is at the top left of each block and percent methylation is at the top right. Black circles indicate methylated CpGs, white circles indicate unmethylated CpGs. SO, superovulation; EC, *in vitro* embryo culture.

Kindsfather Figure S7

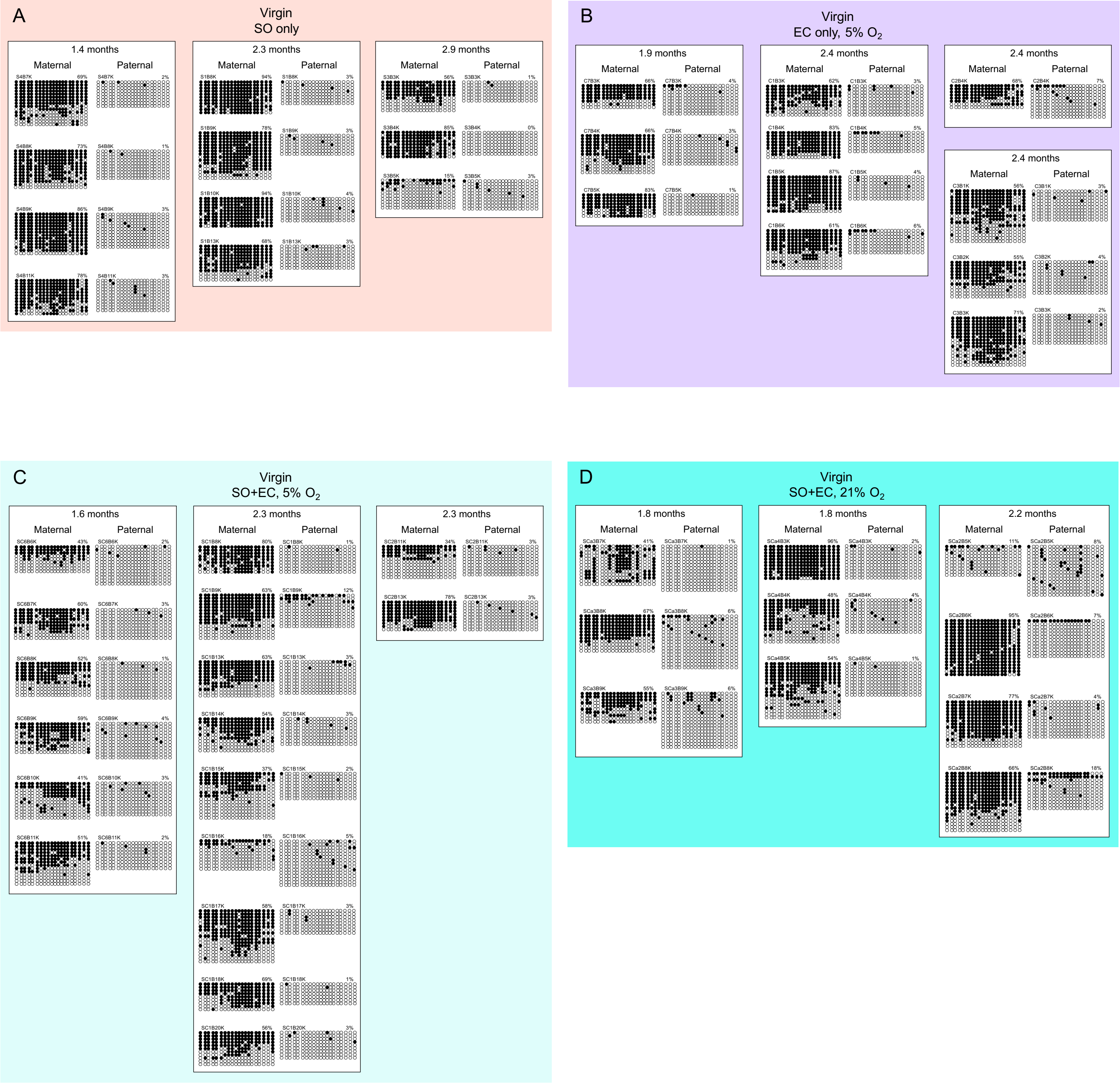

Figure S7. Imprinted methylation maintenance at the normally methylated maternal *Kcnq1ot1* ICR in individual blastocysts from virgin females after ART treatment. (A) SO only; **(B)** EC only with 5% O_2; **(C)** SO+EC with 5% O_2; **(D)** SO+EC with 21% O_2 (n=10-17 embryos; n=3-4 females per treatment group). See Figure S6 for details.

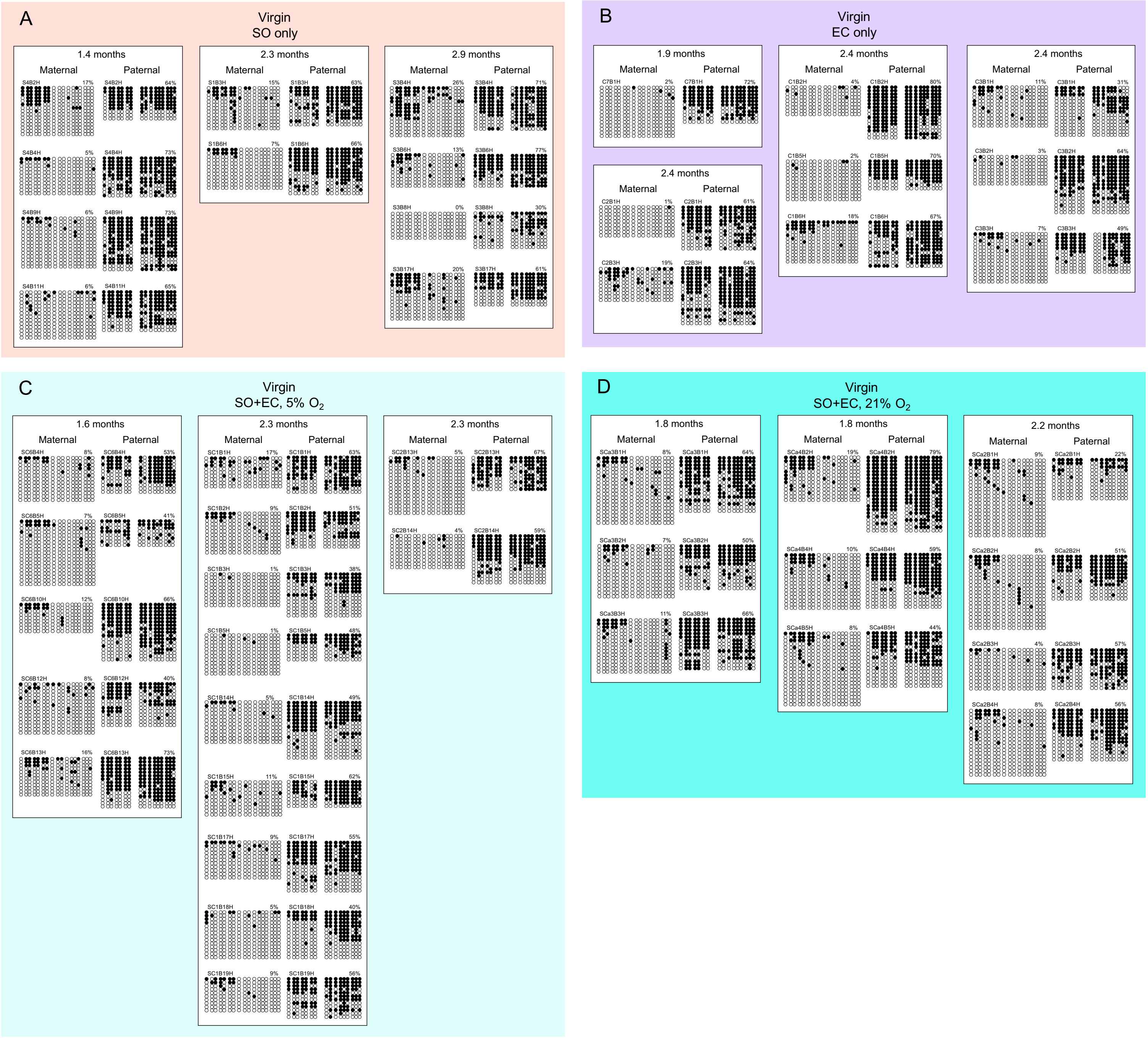

Figure S8. Imprinted methylation maintenance at the normally methylated paternal *H19* ICR in individual blastocysts from virgin females after ART treatment. (A) SO only; **(B)** EC only with 5% O_2; **(C)** SO+EC with 5% O_2; **(D)** SO+EC with 21% O_2 (n=9-16 embryos; n=3 females per treatment group). See Figure S6 for details.

Kindsfather Figure S9

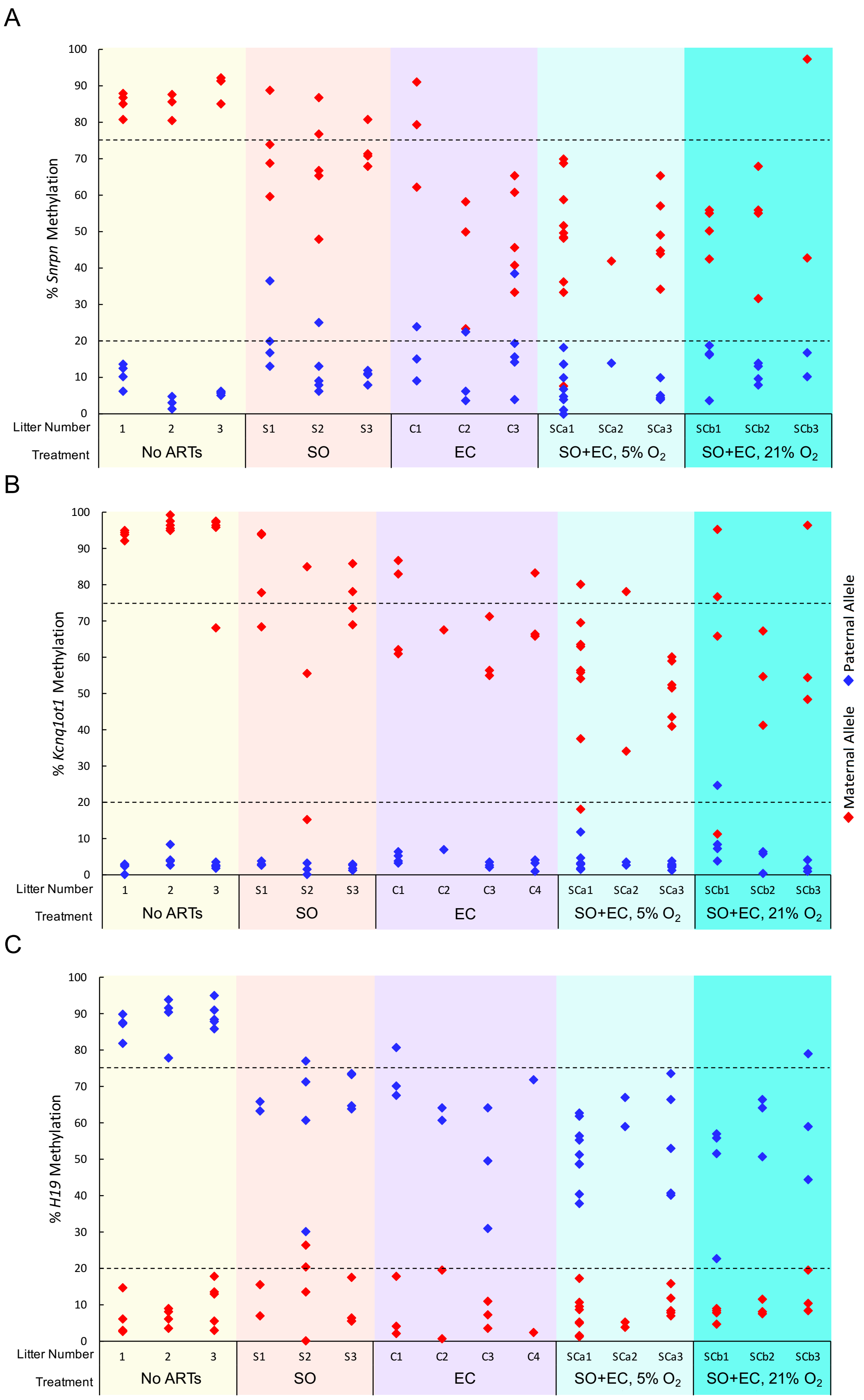

Figure S9. At least one blastocyst from every ART-treated litter lost imprinted methylation on the normally methylated *Snrpn, Kcnq1ot1,* or *H19* ICRs. Diamonds represent the average percent methylation of maternal (red) or paternal (blue) alleles for individual embryos. ARTs, assisted reproductive technologies; SO and S, superovulation; EC and C, embryo culture; SC, superovulation plus embryo culture.

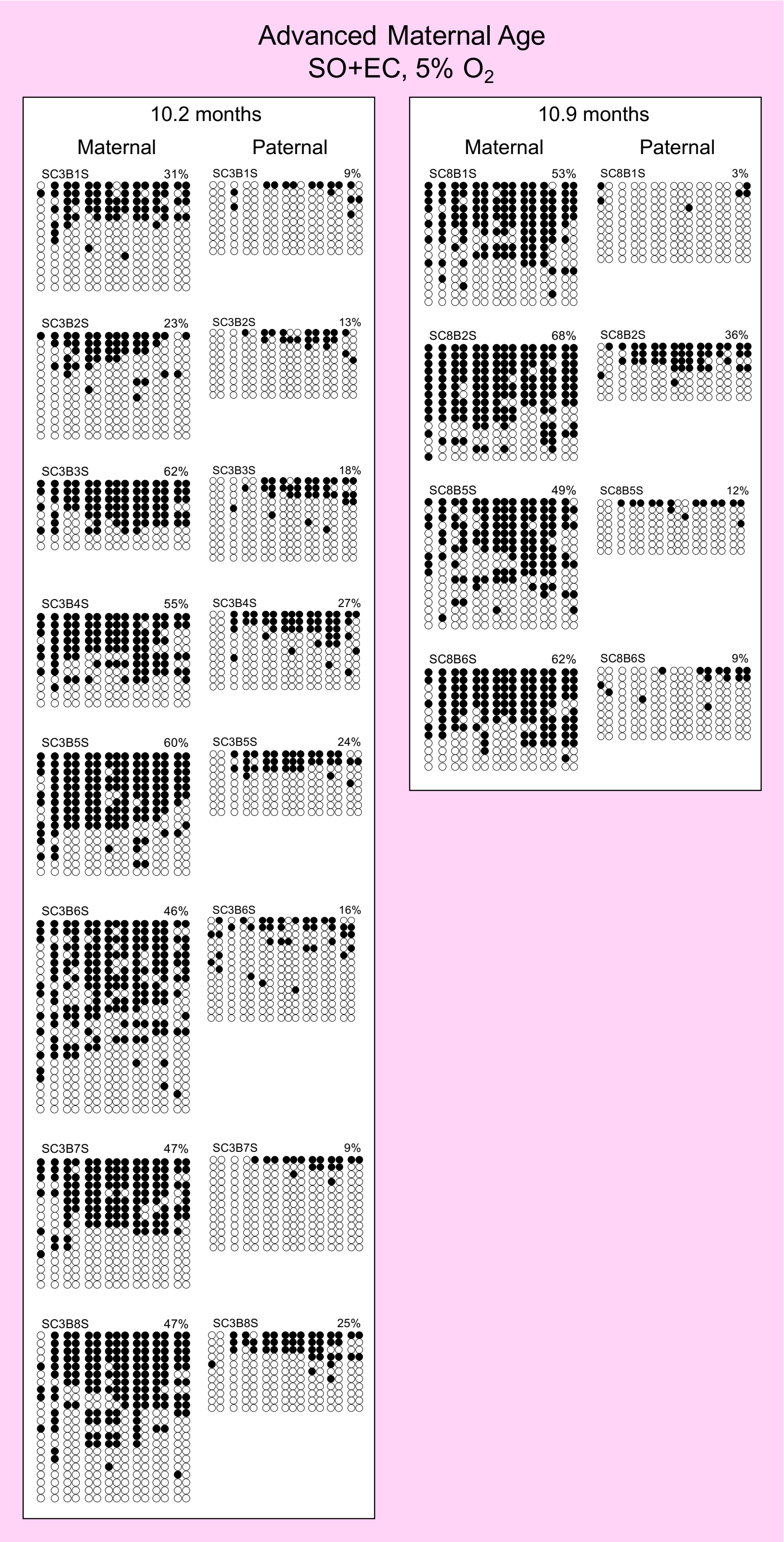

Figure S10. Imprinted methylation maintenance at the normally methylated maternal *Snrpn* ICR in individual blastocysts from advanced maternal age females after ART treatment. Each box encloses embryos analyzed from one female. Each block denotes an individual embryo. Each line represents an individual strand of DNA with the maternal allele [C57BL/6(CAST7)] on the left and the paternal allele (C57BL/6) on the right. Embryo designation is at the top left of each block and percent methylation is at the top right. Black circles, methylated CpGs; white circles, unmethylated CpGs; SC, superovulation plus embryo culture.

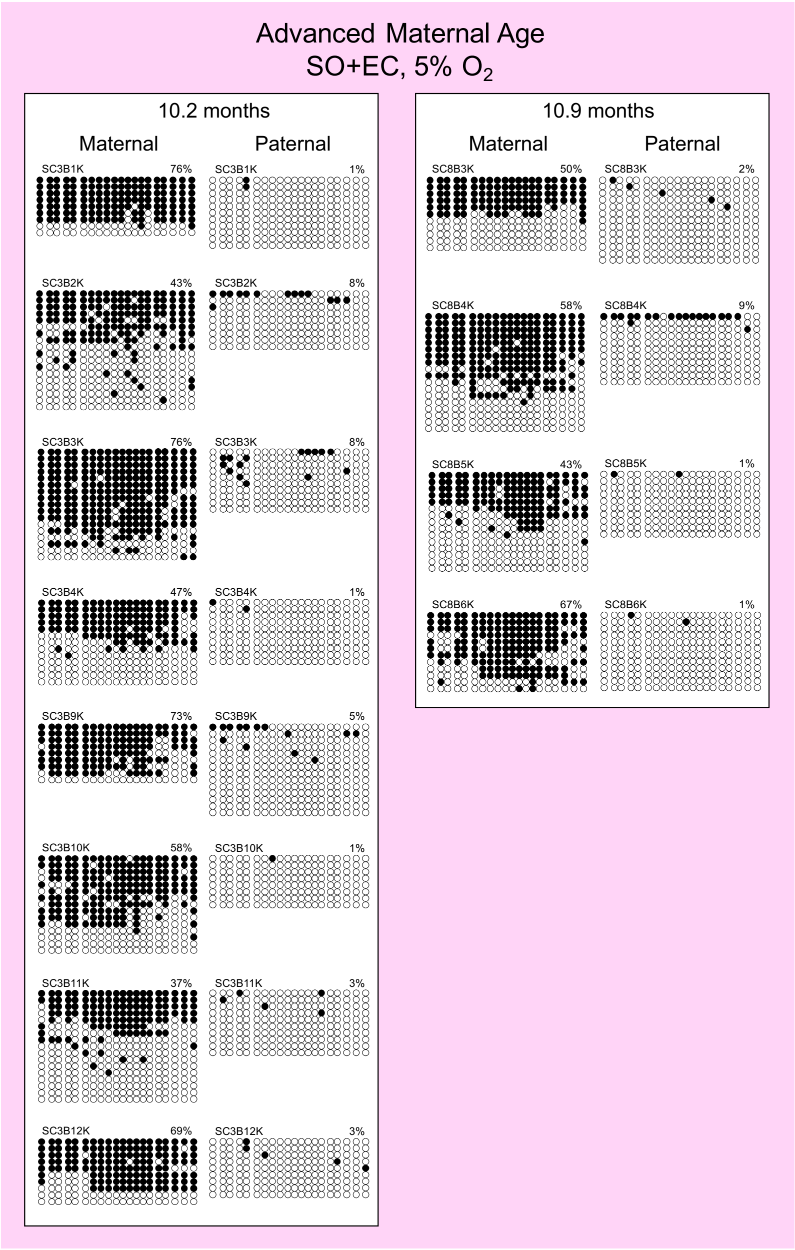

Figure S11. Imprinted methylation maintenance at the normally methylated maternal *Kcnq1ot1* ICR in individual blastocysts from advanced maternal age females after ART treatment. See Figure S10 for details.

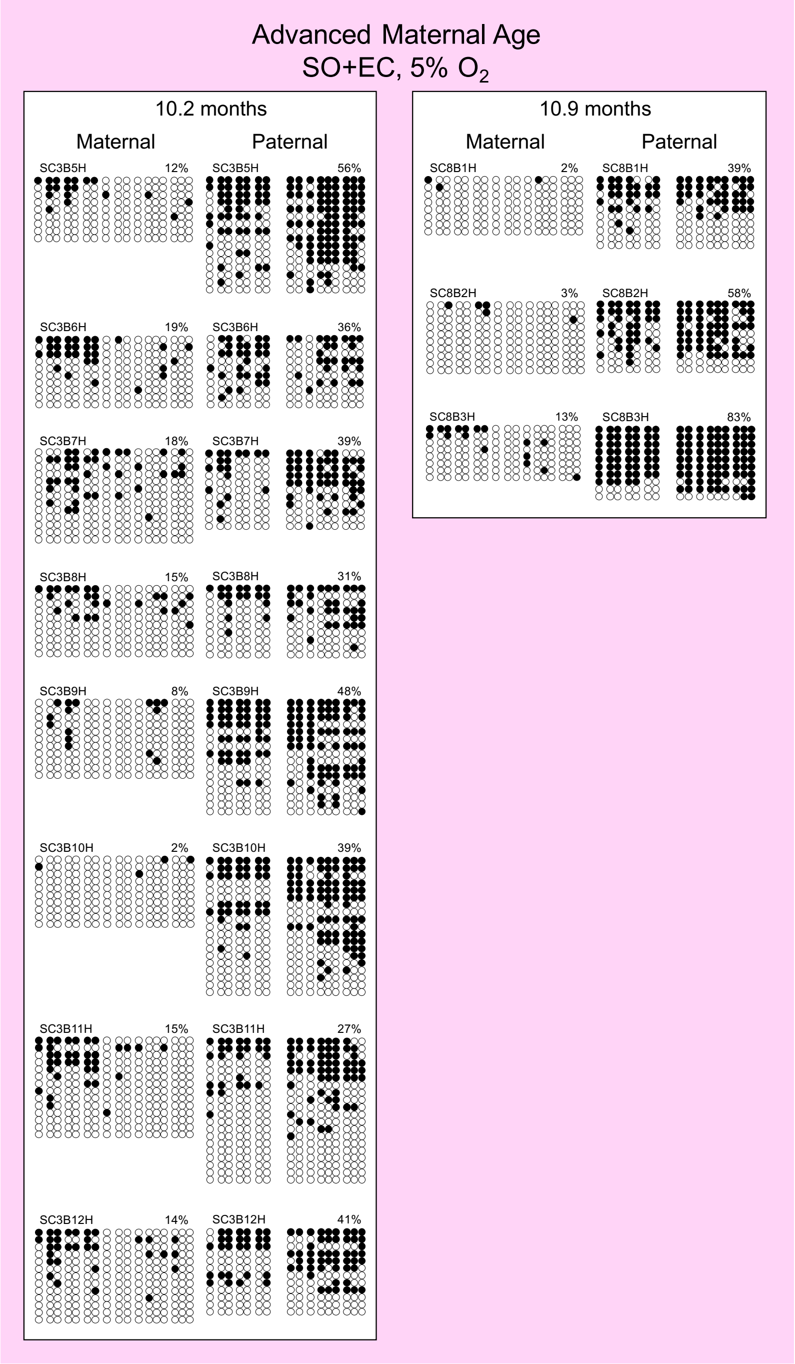

Figure S12. Imprinted methylation maintenance at the normally methylated paternal *H19* ICR in individual blastocysts from advanced maternal age females after ART treatment. See Figure S10 for details.

Table S1: Comparison of treatment groups using Fisher exact test.

Group 1	Group 2	Maternal *Snrpn* ICR p-value	Maternal *Kcnq1ot1* ICR p-value	Paternal *H19* ICR p-value
No ARTs	SO Only	0.00087	0.048	<0.0001
No ARTs	EC Only, 5% O_2	0.00019	0.0017	<0.0001
No ARTs	SO+EC, 5% O_2	<0.0001	<0.0001	<0.0001
SO Only	EC Only, 5% O_2	NS	NS	NS
SO Only	SO+EC, 5% O_2	0.026	0.022	NS
EC Only, 5% O_2	SO+EC, 5% O_2	NS	NS	NS
SO+EC, 5% O_2	SO+EC, 21% O_2	NS	NS	NS

NS, not significant; ICR, imprinting control region; ARTs, assisted reproductive technologies; SO, superovulation; EC, embryo culture

Table S2: Comparison of maternal age and treatment groups using Fisher exact test.

Group 1	Group 2	Maternal *Snrpn* ICR p-value	Maternal *Kcnq1ot1* ICR p-value	Paternal *H19* ICR p-value
No ARTs, Virgin	No ARTs, Advanced Age	NS	NS	NS
No ARTs, Virgin	SO+EC, Virgin	<0.0001	<0.0001	<0.0001
No ARTs, Advanced Age	SO+EC, Advanced Age	<0.0001	0.0001	<0.0001
SO+EC, Virgin	SO+EC, Advanced Age	NS	NS	NS

NS, not significant; ICR, imprinting control region; ARTs, assisted reproductive technologies; SO+EC superovulation and embryo culture.

Table S3: Bisulfite mutagenesis and PCR amplification

Gene	Accession	Position	Primer type	Primer sequence (5' – 3')	Sodium bisulfite incubation time	Annealing temperature
Snrpn	AF081460	2151-2570	OF	TATGTAATATGATATAGTTTAGAAATTAG	3.5 hours	50°C
			OR	AATAAACCCAAATCTAAAATATTTTAATC		
			IF	AATTTGTGTGATGTTTGTAATTATTTGG		50°C
			IR	ATAAAATACACTTTCACTACTAAAATCC		
Kcnq1ot1	AJ271885	141392-141598	OF	GTGTGATTTTATTTGGAGAG	3 hours	52°C
			OR	CCACTCACTACCTTAATACTAACCAC		
			IF	GGTTAGAAGTAGAGGTGATT		52°C
			IR	TACTGAATTCCAAAACCACCCCTACTTCTAT		
H19	U19619	1304-1726	OF	GAGTATTTAGGAGGTATAAGAATT	3.5 hours	50°C
			OR	ATCAAAAACTAACATAAACCCCT		
			IF	GTAAGGAGATTATGTTTATTTTTGG		50°C
			IR	CCTCATTAATCCCATAACTAT		

OF, outer forward; OR, outer reverse; IF, inner forward; IR, inner reverse.
